# Stimuli-Responsive Peptides for Targeted Anticancer Drug Delivery: Current Advances and Future Outlook

**DOI:** 10.3390/pharmaceutics18060732

**Published:** 2026-06-13

**Authors:** Oindrila Palit, Ankita Das, Supriya Bharti, Eirinaios I. Vrettos, Sankarprasad Bhuniya

**Affiliations:** 1 Centre for Interdisciplinary Sciences, JIS Institute of Advanced Studies and Research, JIS University, 51, South Nayabaz, GIP Colony, Santragachi, Nibra, Howrah 711302, West Bengal, India; donapalit20@gmail.com (O.P.); ankitadasmum@gmail.com (A.D.); supriyabharti1474@gmail.com (S.B.); 2 Department of Chemical Biology and Therapeutics, St. Jude Children’s Research Hospital, Memphis, TN 38105, USA

**Keywords:** stimuli-responsive, peptide, hypoxia, ROS, enzymes, apoptosis, tumor, in vivo

## Abstract

Efficient delivery systems are essential for transporting chemotherapeutic agents to target sites, enhancing cellular uptake and reducing off-target side effects. Peptides, owing to their intrinsic biocompatibility and structural tunability, have emerged as promising carriers for delivering labile chemotherapeutics and improving pharmacokinetics and therapeutic outcomes. Along these lines, a wide variety of peptide-based delivery strategies have been developed to achieve desirable pharmaceutical properties for anticancer agents. Particularly, stimuli-responsive peptide-based nanocarriers have attracted high levels of attention due to their ability to exploit overexpressed or tumor-specific stimuli, enabling selective disassembly and controlled drug release within cancer cells. In this review, we highlight recent advances in the development of stimuli-responsive peptide nanocarriers and their applications in anticancer therapy, and discuss key challenges and future directions toward their clinical translation.

## 1. Introduction

Cancer is recognized as one of the deadliest diseases, with a high mortality rate worldwide. Despite its long-standing existence, its intrinsic complexity and heterogeneity pose significant challenges to achieving effective therapeutic outcomes. As early as the sixteenth century, the pioneering physician Paracelsus—a German-Swiss alchemist regarded as the father of modern medicine—employed organic compounds and minerals for the treatment of cancer [1]. Currently, over 200 drugs have been approved by the FDA, with nearly 90% prescribed primarily for palliative care, aiming to stabilize disease progression and improve patients’ quality of life [2]. Regrettably, many chemotherapeutic agents often exhibit limited efficacy while exerting significant cytotoxicity toward healthy cells, raising concerns regarding systemic toxicity, off-target effects, and overall patient safety [3]. The concept of anticancer drug delivery systems emerged in the 19th century and advanced significantly during the 1960s–1970s, aiming to enable targeted delivery, optimize pharmacokinetic properties, and minimize the side effects associated with chemotherapeutic agents [4]. Additionally, drug delivery systems (DDS) enhance cellular uptake, facilitate safe transport of the drug to the target sites, and prolong systemic circulation, thereby improving overall accumulation in cells and tissues. Various nanocarrier systems, including liposomes, solid lipid nanoparticles, dendrimers, polymeric micelles, virus-based nanoparticles, and organic–inorganic hybrid nanocarriers, have been extensively explored for anticancer drug delivery applications. Following Robert Merrifield’s development of solid-phase peptide synthesis (SPPS) in 1963, peptide-based nanocarriers have emerged as promising platforms and have been increasingly utilized in anticancer drug delivery since the 1970s [5,6,7]. Due to their excellent biosafety, biocompatibility, low toxicity, and versatile design strategies, a wide variety of peptides have been extensively exploited as drug delivery systems. It is worth highlighting that approximately 60 peptide therapeutics have reached the market, with hundreds more currently under clinical investigation [8]. Typically, peptides comprising 6 to 50 amino acids are designed and utilized for targeted drug delivery applications [9]. To achieve precise targeted delivery, the peptide backbone is strategically engineered to incorporate stimuli-responsive elements that enable site-specific payload release. Stimuli-responsive peptides are often combined with cleavable linkers that can be selectively activated by external triggers or by endogenous stimuli commonly found in tumor environments. Upon activation, these linkers undergo cleavage or structural disassembly, facilitating controlled release of the anticancer drug at the target site.

In advanced drug delivery systems, peptides are further functionalized with cell-penetrating motifs or cancer-specific ligands to guide the cargo selectively toward tumor cells. The stimuli-responsive segment is designed to be highly sensitive to specific triggers, ensuring efficient and controlled drug release, while the remaining peptide framework serves as a stable carrier for targeted delivery.

**Figure 1:**
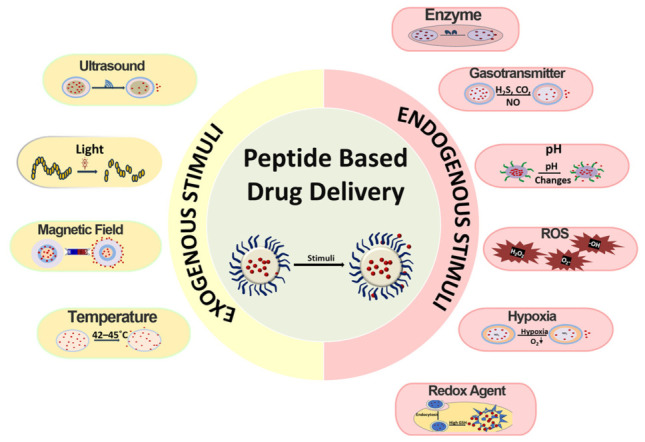
Schematic representation of stimuli-responsive peptide-based drug delivery systems (DDS), categorized into exogenous and endogenous triggers.

In this review, we discuss the design architectures and applications of peptide-based nanocarriers that respond to both external stimuli, such as light, ultrasound, magnetic fields, and temperature, as well as endogenous triggers, including redox conditions (e.g., biothiols), reductase enzymes associated with hypoxia, reactive oxygen species (ROS), enzymes, and gasotransmitters (Figure 1). These systems are systematically categorized, and their advantages, design principles, limitations, and future perspectives are critically outlined.

## 2. External Stimuli-Responsive Peptide

Targeted drug delivery is essential for minimizing off-target cytotoxicity; however, dose optimization is equally critical for achieving rapid and effective therapeutic outcomes. Proper dosing is crucial for maximizing the effectiveness of highly potent anticancer agents while minimizing the risk of drug efflux, whereby excess drug is expelled from target cells, compromising therapeutic effectiveness and negatively affecting surrounding healthy tissues.

Researchers have developed advanced controlled drug release strategies centered on stimuli-responsive systems. These systems are designed to release therapeutic agents in a controlled way, triggered by specific external stimuli such as changes in temperature, pH, or the presence of certain biomolecules [10]. By leveraging these stimuli-responsive mechanisms, drug release can be precisely controlled in both spatial and temporal dimensions, thereby improving therapeutic efficacy while reducing off-target effects. This innovative approach not only improves patient outcomes but also represents a significant advancement in the field of pharmacology and drug delivery technologies. These systems utilize non-invasive or minimally invasive external triggers—such as light, ultrasound, or magnetic fields—to control the release of anticancer drugs from polymeric nanoscaffolds [11]. In the mid-1980s, Langer and co-workers first demonstrated the controlled release of a macromolecule (bovine serum albumin) from a biocompatible polymer system under an oscillating magnetic field [12]. In their approach, small magnetic spheres or cylindrical magnets were embedded in the polymer matrix, which, upon exposure to the oscillating field, enhanced the release rate by approximately 5–10-fold compared to the control [12]. In contemporary times, Kim and co-workers demonstrated temperature-triggered release of indomethacin from poly(NIPAAm-co-BMA). By cycling the temperature between 20 °C and 30 °C in steps, they observed thermally induced polymer swelling, which served as the key mechanism driving drug release [13]. Shozo Miyazaki and colleagues demonstrated a feasible approach for pulsatile drug delivery in diabetic rats by externally controlling insulin release from hydrophilic polymer implants using ultrasound irradiation. Their study showed that insulin-loaded hydrophilic polymers (e.g., EVA copolymers) could achieve on-demand release upon ultrasound exposure, leading to significantly enhanced insulin release and a corresponding reduction in blood glucose levels [14]. Since then, various advanced strategies have been developed to design exogenously stimulated drug delivery systems, utilizing triggers such as temperature, light, magnetic fields, and ultrasound. The following sections describe peptide-based systems engineered to respond to these external stimuli.

### 2.1. Temperature-Sensitive Peptide

Temperature is a unique external stimulus that can trigger drug release from peptide-based carriers through thermally induced structural changes or dissolution. In addition, localized heating in the range of 40–45 °C induces hyperthermia, which can directly damage or kill cancer cells by disrupting protein function and cellular integrity. Therefore, thermally triggered drug delivery offers dual therapeutic advantages: controlled release of chemotherapeutic agents and synergistic anticancer effects arising from hyperthermia, ultimately enhancing the inhibition of tumor cell proliferation. Following the successful development of elastin–peptide conjugates as thermoresponsive anticancer drug carriers [15,16], Al-Ahmady et al. reported a thermoresponsive liposomal system incorporating a temperature-sensitive amphiphilic peptide within the lipid bilayer (Lp-peptide). This design features a leucine zipper peptide sequence [VSSLESKVSSLESKVSKLESKKSKLES-KVSKLESKVSSLESK]-NH_2_, which undergoes structural dissociation above its melting temperature (approximately 40 °C). This transition leads to a disordered conformation, as confirmed by CD spectra. This conformational change destabilizes the lipid bilayer, thereby triggering the release of the encapsulated anticancer drug doxorubicin [17]. They prepared liposomes by incorporating the peptide into thermoresponsive DPPC/DSPC/DSPE-PEG2000 (90:10:5) lipid formulations at varying lipid-to-peptide molar ratios (600:1, 200:1, and 100:1). The resulting liposomes exhibited hydrodynamic diameters in the range of 97–128 nm with low polydispersity, and a phase transition temperature (T_m_) between 41.39 and 42.95 °C (Table 1).

They investigated the effect of peptide anchoring on the temperature responsiveness of vesicles by evaluating the release of doxorubicin (DOX) at 42 °C in 50% serum. Nearly 100% of DOX was released from temperature-sensitive liposomes within the first minute of incubation. In contrast, DPPC/DSPC/DSPE-PEG2000 liposomes displayed a gradual increase in drug release, achieving approximately 80% after 5 min and nearly 100% after 30 min. No significant difference in DOX release was observed between conventional liposomes and Lp-peptide hybrids at lipid-to-peptide ratios up to 200:1. However, at a 100:1 ratio, only about 60% of DOX was released from the Lp-peptide hybrids over 1 h. Cheng et al. developed thermosensitive hydrogels from PEG and poly(γ-ethyl-L-glutamate) through ring-opening polymerization of ELG-NCA with amino-terminated PEG [18]. Concentrated solutions of the resulting copolymers exhibited a sol–gel phase transition in response to temperature, driven by partial dehydration of PEG segments and enhanced packing of the β-sheet-forming PELG domains.

These hydrogels were gradually degraded and eliminated within three weeks following subcutaneous injection in mice, while H&E staining confirmed their biocompatibility for in vivo applications. Paclitaxel (PTX) was incorporated into the hydrogel matrix to function as an in situ depot system for sustained drug release. In vivo antitumor studies demonstrated that PTX-loaded hydrogels effectively suppressed tumor growth (both volume and weight) for up to 21 days, without causing significant organ toxicity. Kim et al. developed a thermosensitive, tumor-targeted liposomal system for delivering anticancer drugs by incorporating elastin-like polypeptide (ELP) into DPPC-based liposomes and introducing a cRGD ligand to target the αvβ3 integrin [19]. Doxorubicin (DOX)-loaded liposomes (RELs) were prepared with an average size of ~181 nm. The RELs exhibited efficient temperature-triggered release, achieving ~75% and ~83% DOX release at 42 °C and 45 °C, respectively, while maintaining stability for up to 12 h. Cellular uptake studies showed significantly enhanced internalization (8–10 fold) in αvβ3-expressing U87MG and HUVEC cells compared to non-targeted systems. Confocal imaging confirmed DOX release under mild hyperthermia (42 °C), with nuclear localization of DOX. In vitro and in vivo studies demonstrated enhanced cytotoxicity and ~5-fold higher tumor accumulation, highlighting the potential of this thermosensitive, targeted liposomal system for improved cancer therapy.

Ryu et al. engineered an elastin-like polypeptide (ELP) by appending a Bac cell-penetrating peptide (RRIRPRPPRLPRPRPRPLPFPRPG) at the C-terminus and a p21Waf1/Cip1-derived peptide (GRKRRQTSMTDFYHSKRRLIFSKRKP) or its scrambled control at the N-terminus [20]. Leveraging the thermoresponsive behavior of ELP, mild local hyperthermia was used to enhance tumor targeting and delivery. The p21–ELP construct demonstrated significant anticancer activity in pancreatic cancer models and, when combined with gemcitabine, exhibited enhanced cytotoxicity in vitro and effectively inhibited tumor growth in vivo. Recently, Zhang et al. addressed key limitations of conventional hydrogels—such as poor mechanical strength and rapid dissolution—by incorporating gelatin into a pH-responsive peptide (Ac-FALNLAKD-NH_2_) to form gelatin/peptide composite hydrogels (Gel/PEP) for controlled delivery of the anticancer drug chelerythrine (CHE) [21]. Structural analyses using circular dichroism and FTIR indicated a mixed secondary structure comprising β-sheets (44.7%) and random coils (55.3%). Rheological and SEM studies revealed a dense nanofibrous network, contributing to enhanced mechanical stability and injectability. The Gel/PEP system achieved >97% CHE encapsulation at a gelatin-to-peptide ratio of 7:1 and exhibited effective pH- and temperature-responsive drug release behavior.

Among the reported hyperthermia-based drug delivery systems, the platform developed by Kim et al. exhibited one of the most impressive anticancer performances, achieving approximately 83% DOX release and nearly fivefold higher tumor-specific accumulation [19]. Overall, hyperthermia-assisted peptide-based delivery systems represent a promising strategy for site-specific anticancer therapy by combining controlled drug release with localized thermal treatment, thereby enhancing therapeutic efficacy (Table 1). Nevertheless, further preclinical and clinical investigations are required to facilitate their successful translation into clinical practice.

### 2.2. Light-Activated Peptide

**Figure 2:**
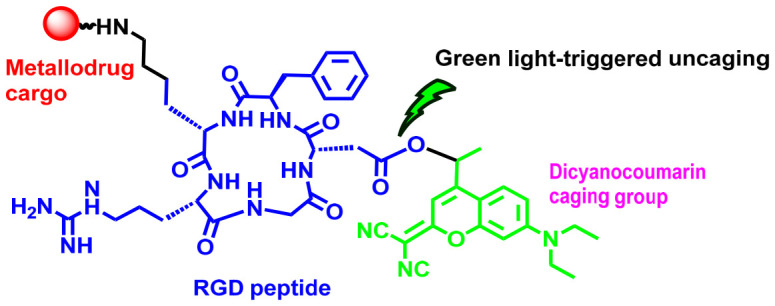
Photo-triggered metallodrug delivery system. Figure adapted with permission from [23]. Copyright 2016, American Chemical Society.

Light is a highly attractive external stimulus for non-invasive biological applications, offering excellent spatial and temporal precision with minimal toxicity. Photocaged compounds remain biologically inactive until light exposure restores their structure and function. This strategy is particularly advantageous in cancer therapy, where light-activatable peptide-based nanocarriers enable precise, on-demand drug activation. Moreover, it allows real-time control over treatment location and duration, facilitating therapeutic monitoring. Such selective activation of masked theranostic systems provides a promising approach to overcoming drug resistance in cancer. Mei and co-workers developed photoresponsive cell-penetrating peptides (CPPs: CGRRMKWKK) to enable NIR-triggered intracellular delivery of liposomes [22]. The positive charges of lysine residues in CPPs were temporarily masked with a two-photon NIR-responsive protecting group, (4,5-dimethoxy-2-nitrophenyl)methanamine, generating photosensitive peptides (PSPs). These PSPs were conjugated to DSPE via a PEG linker to form modified liposomes (PSP-L). Upon NIR irradiation (λ_em_ = 740 nm, 3.48 × 10^12^ photon s−^1^) at tumor sites, the protecting groups were cleaved, restoring CPP activity and promoting rapid cellular uptake of the liposomes. The PSP-L system loaded with Navelbine exhibited suitable physicochemical properties, enhanced cellular uptake, and increased cytotoxicity in MCF-7 cells under NIR illumination. Moreover, NIR-triggered activation of PSP-L resulted in significantly improved antitumor efficacy in an MCF-7 xenograft mouse model compared to non-modified liposomes. Marchán and co-workers synthesized a coumarin-caged cyclic RGD peptide and investigated its photochemical properties, demonstrating efficient uncaging under biologically compatible green light (Figure 2) [23].

This was achieved using a dicyanocoumarin derivative (DEAdcCE) to protect the carboxyl group of the aspartic acid side chain, selected for its compatibility with Fmoc-tBu SPPS and high photolysis efficiency. The incorporation of a methyl group near the ester linkage in the coumarin moiety, along with additives such as HOBt or Oxyma, effectively minimized aspartimide-related side reactions. Furthermore, a bioconjugate of the caged peptide with ruthenocene, used as a model metallodrug, was successfully synthesized. The efficient green-light-triggered activation of both the peptide and its ruthenocene conjugate highlights the potential of DEAdcCE-caged peptides as selective carriers for photocontrolled anticancer therapy.

Zhao and co-workers developed a biocompatible block copolymer, PEO_114_-b-P(LGA_0.62_-co-COU_0.38_)_34_, incorporating 6-bromo-7-hydroxycoumarin-4-ylmethyl groups for near-infrared (NIR)-triggered drug delivery [24]. The micelles were disrupted under 794 nm NIR irradiation via two-photon absorption, caused by the removal of coumarin groups. This change affected the hydrophilic-hydrophobic balance and destabilized the micellar structure in aqueous media. This NIR-triggered mechanism enabled controlled release of encapsulated drugs, including rifampicin and paclitaxel (PTX). Notably, NIR-irradiated micelles exhibited sustained PTX release (~50% over 145 h), whereas negligible release was observed in the absence of irradiation, highlighting the effectiveness of the light-responsive system. Similarly, Adler-Abramovich and co-workers developed NVOC-FF nanofiber hydrogels that undergo photocleavage upon exposure to 365 nm UV light, generating unmasked FF peptides that disrupt the supramolecular assembly [25]. This process leads to hydrogel degradation and efficient release of the encapsulated drug. Similarly, Cheng and coworkers designed a light-responsive helical polypeptide by incorporating 3,4-dimethoxy-2-nitrobenzyl groups [26]. This system exhibits strong DNA-binding affinity under dark conditions, but undergoes conformational disruption upon UV irradiation, resulting in unwinding of the helical structure and subsequent release of the bound genetic material.

**Figure 3:**
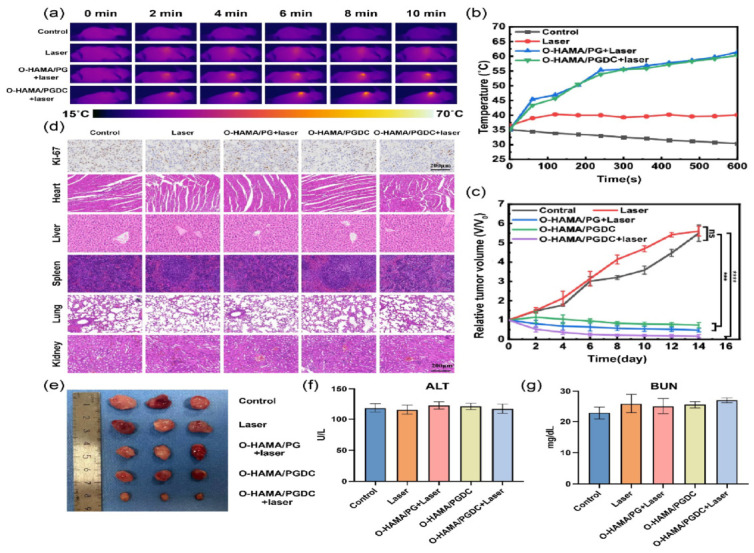
In vivo evaluation of the O-HAMA/PGDC hydrogel included photothermal performance, antitumor efficacy, and systemic biosafety: (**a**) infrared thermal imaging of tumor-bearing mice under 808 nm laser irradiation at different time points; (**b**) corresponding temperature elevation profiles at tumor sites; (**c**) relative tumor volume changes across treatment groups; (**d**) Ki-67 immunohistochemical staining of tumor tissues and H&E staining of major organs (heart, liver, spleen, lung, and kidney); (**e**) photographs of excised tumors after treatment; and (**f**,**g**) serum biochemical analysis of ALT and BUN levels. Figure adapted with permission from [27]. Copyright 2026, Springer nature.

Wei and his team developed biometallic peptide–drug conjugates (PGDCs) with peptide nanofibers, gold nanoparticles, and doxorubicin, integrated into photothermal and chemotherapeutic dual-network hyaluronic acid hydrogels for breast cancer treatment [27]. The hydrogel, created by mixing oxidized methacrylated hyaluronic acid with PGDCs and quickly crosslinked under UV light, allowed localized drug deployment in just 90 s. These hydrogels demonstrated pH-responsive drug release in tumor microenvironments and effective photothermal performance under near-infrared (NIR) irradiation. In vivo studies using a 4T1 tumor model in BALB/c nude mice showed a significant temperature increase (up to 62 °C) with NIR light (Figure 3a), sufficient to damage tumor cells while enabling localized DOX release, minimizing systemic toxicity (Figure 3b–d). The PTT/CT group exhibited the greatest tumor inhibition, with a normalized relative tumor volume of 0.20 ± 0.11, significantly lower than the control and single treatment groups (*p* < 0.001). This enhanced effectiveness was due to heat-induced apoptosis and increased DOX uptake through hyperthermia-enhanced membrane permeability. Notably, ALT and blood urea nitrogen levels were similar to those of the control, indicating minimal organ toxicity (Figure 3f,g).

Although several studies [24,25,26] have demonstrated NIR light-triggered peptide-polymer disassembly for the release of chemotherapeutic agents, Wei and co-workers reported a bimetallic peptide–drug conjugate system that combines light-triggered DOX release with photothermal hyperthermia. Upon NIR irradiation, the system effectively eradicated tumors through apoptosis while minimizing toxicity to normal tissues. This study emphasizes the promise of peptide-based platforms that combine targeted payload delivery with light-induced hyperthermia, offering a potential strategy for developing next-generation drug delivery systems. While ongoing studies continue to improve therapeutic performance, additional preclinical and clinical data are required to establish their safety, efficacy, and translational potential for practical applications.

### 2.3. Ultrasound-Sensitive Peptide

**Figure 4:**
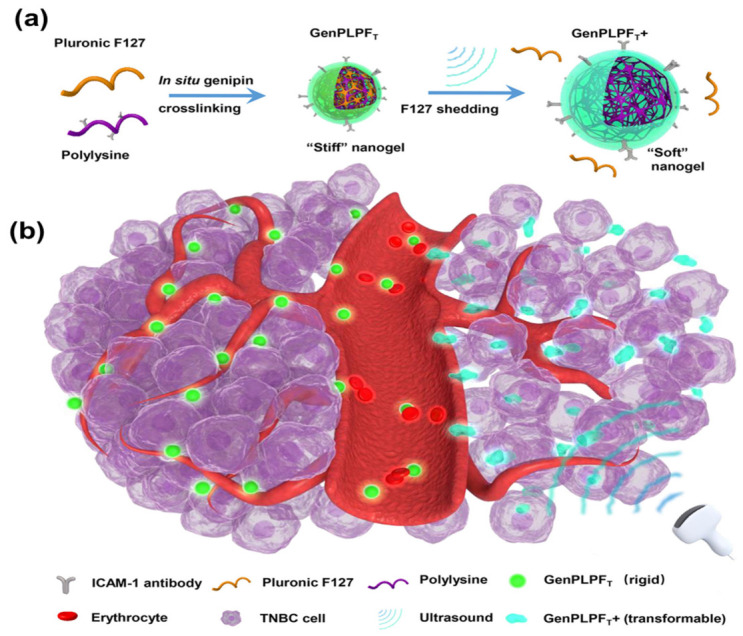
Schematic presentation of polylysine- and Pluronic F127-functionalized drug delivery system. (**a**) Polylysine and Pluronic F127 nanogel and rupture under sonication; (**b**) Sonication triggers the polylysine rupture in TNBC cells. Figure adapted with permission from [33]. Copyright 2022, American Chemical Society.

Ultrasound (US) is a gentle and appealing external stimulus that provides precise spatiotemporal control over cargo delivery to disease sites while minimizing damage to healthy tissues [28,29,30]. It also provides several advantages, including non-invasiveness, widespread clinical availability, absence of ionizing radiation, and the ability to modulate tissue penetration with relative ease. In this context, Han et al. developed paclitaxel (PTX)-loaded thiolated human serum albumin (tHSA) nanoparticles (PTX-tHSA-NPs) conjugated with microbubble (MB) complexes, denoted as PTX-tHSA-NPs-MBs, for high-intensity focused ultrasound (HIFU)-triggered drug delivery [31]. In vitro, the HIFU-treated system (PTX-tHSA-NPs-MBs, HIFU+) showed enhanced nanoparticle accumulation due to sonoporation and mechanical effects. In vivo, this group exhibited the greatest antitumor efficacy, yielding the smallest tumor size and lowest tumor weight (0.16 g). Wooley and co-workers synthesized monomethoxy monoamino-terminated poly(ethylene glycol)-based block copolymers, mPEG-b-P(Ala–Gly–Ile) [32]. The polypeptide segments predominantly adopted β-sheet structures, enabling a thermally induced sol–gel transition and sustained release of naproxen over approximately six days. Sun et al. developed a genipin-crosslinked nanogel composed of polylysine and Pluronic F127, further functionalized with ICAM-1 antibody for tumor targeting and loaded with epirubicin (GenPLPF/EPI) (Figure 4) [33]. Upon external ultrasound stimulation, efficient drug release was achieved, leading to significant inhibition of triple-negative breast cancer (MDA-MB-231) cells. In vivo studies demonstrated that ultrasound-triggered treatment (GenPLPF/EPI+) reduced plasma half-life and led to significant decreases in tumor volume and size.

Jiang et al. developed a luminescent agent–peptide aggregation-inducing hybrid nanoplatform (PAHN-1; BP-CFFFVLKLAKLAKDEVDAKRGARSTA) for targeted delivery of bipyrene (BP) [34]. This multifunctional system integrates several distinct modules: the AKRGARSTA sequence targets neuropilin-1 (NRP-1), which is overexpressed in cancer cells, facilitating selective accumulation and cellular internalization. Following uptake, the CFFFVLKLAKLA segment disrupts mitochondrial integrity and activates apoptotic signaling, including upregulation of caspase-3.

The DEVD motif serves as a caspase-3-responsive cleavage site, enabling partial removal of the hydrophilic shell and promoting in situ self-aggregation of the peptide residues. Upon ultrasound irradiation, BP aggregates within mitochondria, leading to enhanced reactive oxygen species (ROS) generation. This cascade triggers apoptosis and significantly inhibits tumor growth in the TNBC 4T1 tumor and its metastasis. Overall, this study presents a sophisticated, stimuli-responsive platform that integrates targeting, self-assembly, and ultrasound activation, offering a promising strategy to enhance the efficacy of sonodynamic therapy (SDT).

**Figure 5:**
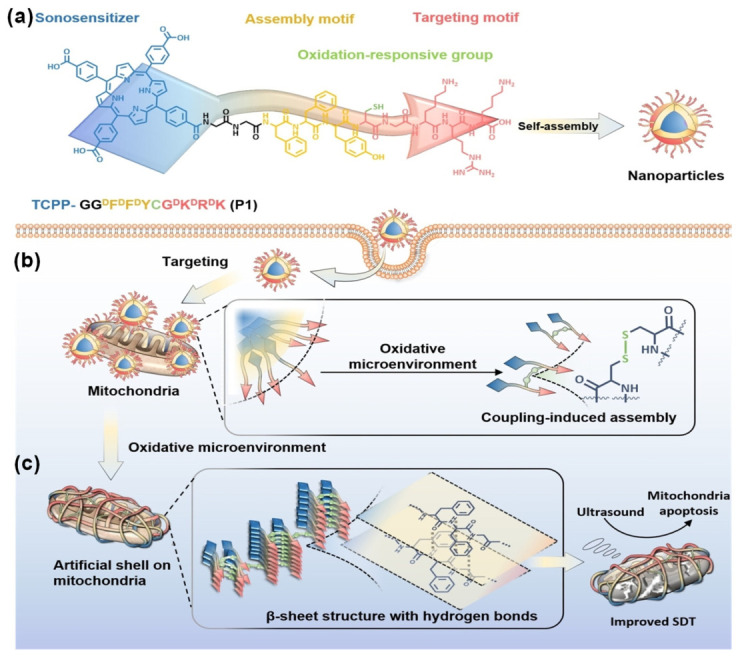
Schematic illustration of the coupling-induced assembly (CIA) strategy for constructing an artificial mitochondrial shell in tumor cells: (**a**) oxidation-responsive peptide–porphyrin conjugates (PPCs) initially self-assemble into nanoparticles, which are internalized via endocytosis and subsequently target mitochondria; (**b**) within the oxidative mitochondrial environment, thiol groups in P1 undergo coupling to form dimers, which further stack into covalently crosslinked nanofibers, generating a stable artificial shell through multivalent interactions; (**c**) upon ultrasound (US) irradiation, the porphyrin units produce abundant reactive oxygen species (ROS), leading to effective induction of apoptosis in tumor cells. Figure adapted with permission from [35]. Copyright 2024, Wiley-VCH GmbH.

Later, Wang and colleagues developed a coupling-induced assembly (CIA) strategy to construct an artificial mitochondrial shell for enhancing sonodynamic therapy (SDT) using a peptide–porphyrin conjugate (P1: TCPPGGDFDFDYCGDKDRDK) (Figure 5) [35].

This multifunctional system comprises three key modules: a mitochondria-targeting and oxidation-responsive segment (CGDKDRDK), a self-assembling unit (FFY), and a porphyrin-based sonosensitizer (TCPP). In the oxidative tumor microenvironment, thiol groups in P1 undergo oxidation to form disulfide-linked dimers, which subsequently self-assemble into covalently crosslinked nanofibers with increased structural rigidity. These nanofibers exhibit enhanced mitochondrial binding via multivalent interactions, forming a stable artificial shell. Upon ultrasound irradiation, the generated ROS disrupt mitochondrial membrane potential, inducing apoptosis. This design avoids premature dissociation of functional groups and ensures structural stability under physiological conditions, ultimately leading to effective mitochondrial disruption and significant tumor growth inhibition in vivo.

Among the various exogenous stimuli explored for peptide-based drug delivery, sonodynamic therapy has demonstrated particularly promising therapeutic efficacy against triple-negative breast cancer (TNBC). Wang and co-workers [35] developed a porphyrin-conjugated peptide system (P1: TCPPGGDFDFDYCGDKDRDK) that preferentially accumulated in mitochondria and induced mitochondrial damage in cancer cells, resulting in a 73.1% reduction in tumor volume in vivo. Similarly, Jiang and colleagues [34] reported an ultrasound-responsive ROS-generating peptide platform, PAHN-1, which triggered mitochondrial dysfunction. In a 4T1 TNBC mouse model, ultrasound irradiation limited tumor growth to only 1.96-fold of its initial volume, whereas untreated tumors increased by approximately 9.42-fold. Notably, treated mice survived for nearly two months after therapy.

Among ultrasound-triggered drug delivery systems, the ICAM-1 antibody-conjugated epirubicin-loaded nanoplatform (GenPLPF/EPI) developed by Sun and co-workers exhibited the most remarkable antitumor performance, achieving 95.57% tumor suppression after 10 days of treatment [33]. Biodistribution studies further confirmed minimal toxicity to major organs. The superior efficacy is likely attributable to antibody-mediated tumor targeting, which enhances drug accumulation at the tumor site. Overall, these findings suggest that chemotherapeutic payload-based sonodynamic systems may offer greater therapeutic benefits than ROS-generating platforms alone.

### 2.4. Magnetic Field Stimulated Peptide

**Figure 6:**
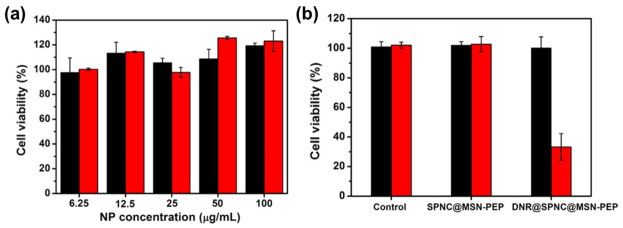
(**a**) Cytotoxicity of SPNC@MSN-PEP (black) and DNR@SPNC@MSN-PEP (red) in PANC-1 cells at varying concentrations (6.25–100 μg/mL). (**b**) Cell viability at 50 μg/mL with and without AMF exposure (control: no treatment). AMF induced only localized heating from the magnetic core without increasing medium temperature, resulting in negligible hyperthermia-induced cytotoxicity. Figure adapted with permission from [42]. Copyright 2019, American Chemical Society.

Paramagnetic nanoparticles inherently respond to an external magnetic field; modulation of the field can induce localized heat generation, which is effective for cancer cell ablation and can also trigger controlled fragmentation of nanocarriers, enabling targeted drug release with minimal disturbance to the surrounding physiological environment [36]. Magnetic nanoparticles (MNPs), typically composed of iron, nickel, or their oxides, display superparamagnetic properties at the nanoscale. In particular, superparamagnetic iron oxide nanoparticles (SPIONs) have gained significant attention due to their multifunctionality. They can be directed and accumulated at target sites using magnetic fields, offer a large surface area for loading therapeutic agents, and efficiently convert electromagnetic energy into heat under an alternating magnetic field. This magnetically induced hyperthermia not only leads to cancer cell destruction but also enhances drug release and triggers apoptosis [37,38,39]. Scarberry and co-workers developed magnetic cobalt spinel ferrite nanoparticles coated with biocompatible polygalacturonic acid and functionalized with peptides targeting EphA2 receptors overexpressed in ovarian cancer cells (Hey and BG-1). These magnetic nanoparticle–peptide conjugates enabled selective capture and extraction of malignant cells under an applied magnetic field [40]. Effective targeting allowed isolation of cancer cells from flow systems in vitro and from the peritoneal cavity in vivo. The successful removal of metastatic cells from both the circulation and the abdominal cavity highlights the potential of this approach as a dialysis-like therapeutic strategy, with prospects for improving long-term outcomes in ovarian cancer patients. Hilt and colleagues developed dextran-coated iron oxide nanoparticles with the TAT cell-penetrating peptide. When A549 and H358 cells internalized these nanoparticles, they were activated by an alternating magnetic field (AMF) [41]. The combination of TAT and AMF increased reactive oxygen species generation compared to nanoparticles alone. Although AMF did not significantly enhance lysosomal permeabilization or mitochondrial depolarization, there was a trend toward increased apoptosis through Caspase 3/7 activation in cells treated with TAT-functionalized nanoparticles under AMF. Ruan et al. developed a drug delivery system using self-assembling peptide-functionalized core–shell mesoporous silica nanoparticles [42]. The core of the system was composed of superparamagnetic manganese- and cobalt-doped iron oxide. A peptide, Boc−Phe−Phe−Gly−Gly−COOH, was covalently attached to the silica shell using 1-ethyl-3-(3-dimethylaminopropyl) carbodiimide hydrochloride and N-hydroxysulfosuccinimide sodium salt. This peptide-based nano self-assembles at physiological temperatures (37 °C) to seal the pore openings and disassembles at an elevated temperature of 50 °C. This property enables controlled drug release of fluorescein and daunorubicin when exposed to heat or an alternating magnetic field. In vitro tests demonstrated significant cytotoxicity against pancreatic carcinoma cells (PANC-1) when activated by the magnetic field, whereas control particles without drugs exhibited no cytotoxicity (see Figure 6a,b).

Lim and colleagues developed Dox-loaded Magnetic Coacervates (DMCs) composed of biomimetic peptide microdroplets and magnetic nanoparticles (MNPs) for thermo-chemotherapy [43]. The controlled release of Dox is triggered by an external Alternating Magnetic Field (AMF) at 45 °C, leading to dual hyperthermia and chemotherapy effects. In vitro studies indicate that DMCs are cytocompatible and effectively induce cell death in HepG2 liver cancer cells more than either hyperthermia or chemotherapy alone. In this context, Lopez-Lopez and co-workers developed biocompatible and biodegradable hydrogels composed of Fmoc-diphenylalanine (Fmoc-FF) and Fmoc-arginine–glycine–aspartic acid (Fmoc-RGD) peptides, incorporating magnetic nanoparticles (MNPs) [44]. These hydrogels exhibited excellent biocompatibility, negligible toxicity, improved mechanical stability, and high injectability, making them promising candidates for minimally invasive in vivo biomedical applications. Qiu and colleagues developed an M2 macrophage-targeted peptide (M2pep)-functionalized superparamagnetic iron oxide nanoparticle (SPIO) for MRI-guided magnetic hyperthermia (MHT) in an aggressive breast cancer (4T1 cells) mouse model [45]. The multifunctional SPIO-M2pep, with a hydrodynamic diameter of 20 nm, demonstrated efficient targeting, high transverse relaxivity (149 mM^−1^s^−1^), and effective MHT in vitro. In vivo studies showed that SPIO-M2pep-based MRI could monitor nanoparticle distribution in tumors and optimize MHT timing, leading to significant reductions in tumor volume and pro-tumoral M2 TAMs. Recently, Zhou et al. reported tumor-specific delivery of magnetic nanoparticles (MNPs) functionalized with tumor-homing peptides PL1 (PPRRGLIKLKTS) and PL3 (AGRGRLVR), enabling dual-targeted accumulation in solid tumors for magnetic hyperthermia [46]. In vitro studies under an alternating magnetic field demonstrated that PL3-MNPs effectively induced cell death in human glioblastoma (U87MG) cells via hyperthermia.

Among the reported magnetic nanoparticle-based peptide delivery systems, most studies have been limited to in vitro evaluations, with only a few investigating in vivo biodistribution [41,42,43,44,45]. Notably, Scarberry and co-workers demonstrated in vivo tumor-selective release of fluorescein diacetate from magnetic cobalt spinel ferrite nanoparticles coated with biocompatible polygalacturonic acid and functionalized with EphA2-targeting peptides under an alternating magnetic field. Nevertheless, compared with magnetic field-responsive systems, ultrasound-triggered peptide-based drug delivery platforms have been more extensively validated in vivo and have shown superior therapeutic outcomes [40]. These findings highlight the strong translational potential of ultrasound-mediated drug release, although further research is required to develop more precise, efficient, and clinically viable exogenous stimulus-responsive delivery strategies.

The exogenous stimuli-responsive peptide-based drug delivery systems outlined in Table 2 highlight significant therapeutic outcomes. While also emphasizing the need for further research, particularly in technological areas such as precise temperature control and advanced photolytic techniques. These improvements are essential for achieving more efficient targeted drug delivery and enhanced therapeutic efficacy. Ultrasound-triggered strategies, together with magnetic nanoparticle-based hyperthermia, have demonstrated considerable promise. However, controlling temperature selectively within the tumor region remains a major challenge, as tumor tissues also contain a substantial population of normal cells. Therefore, the selective eradication of cancer cells while minimizing damage to healthy tissues remains one of the central challenges in cancer therapy.

## 3. Endogenous Stimuli-Responsive Peptide

Tumor-promoting signals are initiated early in cancer progression, for example, through activation of protein kinase C by phorbol esters. In addition, epigenetic alterations, such as hypermethylation of the DNA repair enzyme O6-methylguanine DNA methyltransferase (MGMT), lead to its silencing and impaired DNA repair, a hallmark of carcinogenesis. MicroRNAs, short noncoding RNAs (~20 nucleotides), further regulate gene expression by binding to mRNA, causing degradation or translational inhibition, thereby influencing tumor growth and metastasis [47].

Collectively, these molecular events disrupt cellular communication, cell cycle regulation, adhesion, extracellular matrix organization, and signaling pathways. This dysregulation drives abnormal expression of biomolecules, including enzymes, reactive oxygen species (ROS), and gasotransmitters, ultimately promoting uncontrolled proliferation and metastatic progression. These aberrantly expressed, disease-specific molecules are recognized as cancer biomarkers for diagnosis. Moreover, they can be exploited as triggers for targeted drug release from nanodevices, offering effective strategies to suppress or eradicate cancer. This subsection highlights recent advances in stimuli-responsive peptide-based nanocarriers for targeted anticancer drug delivery, with a particular focus on endogenous triggers such as glutathione (GSH), pH, reactive oxygen species (ROS), gasotransmitters (e.g., NO and H_2_S), enzymes, and hypoxia.

### 3.1. GSH-Stimulated Peptide

**Figure 7:**
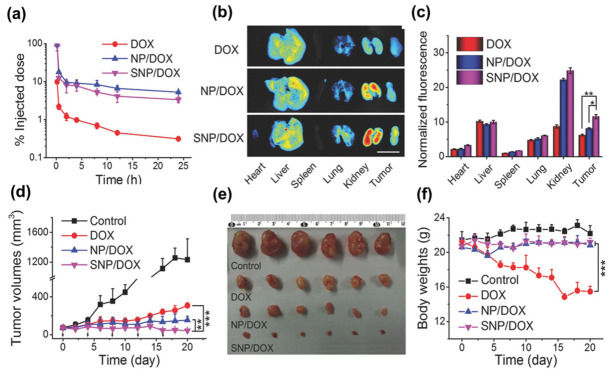
In vivo evaluation of pharmacokinetics, biodistribution, and antitumor efficacy of NP/DOX and SNP/DOX in an A549 lung cancer xenograft model: (**a**) Pharmacokinetic profiles in Sprague–Dawley rats (5 mg kg^−1^ DOX). (**b**) Biodistribution in tumor-bearing mice at 12 h post-injection (scale bar: 0.5 cm). (**c**) Quantitative DOX accumulation in organs and tumors. (**d**) Tumor growth inhibition following treatment with PBS, free DOX, NP/DOX, or SNP/DOX. (**e**) Excised tumor images. (**f**) Body weight changes during treatment. Data are presented as mean ± SD (*n* = 3 for (**a**,**c**); *n* = 6 for (**d**,**f**); * *p* < 0.05, ** *p* < 0.01, *** *p* < 0.001). Figure adapted with permission from [53]. Copyright 2017, Wiley-VCH GmbH.

Glutathione (GSH) is a highly abundant intracellular coenzyme that plays a crucial role in maintaining cellular redox homeostasis. It is predominantly synthesized and localized in the cytosol, where nearly 80% of total GSH is retained to regulate redox balance. Only 10–15% GSH in the mitochondria and about 2–5% GSH in the nucleus [48]. In cancer cells, intracellular GSH levels (5–20 mM) are significantly elevated, often 10–100 times higher than those in normal cells, thereby promoting enhanced proliferation and multidrug resistance [48]. In contrast, extracellular GSH concentrations remain in the micromolar range (approximately 2–20 μM) [49]. This pronounced redox gradient is a key factor enabling the selective intracellular activation of GSH-responsive drug delivery systems (DDSs) in tumor cells [50,51,52]. Chen and co-workers developed a peptide-conjugated shell-stacked nanoparticle (SNP) system with a stable core and thick shell for efficient drug delivery. The core consists of a disulfide-crosslinked nanogel (PZLL10−P(LP7-co-LC5)), while the shell is formed by DMMA-modified mPEG-b-PLL (Shell-DMMA) exhibiting charge-reversal behavior [53]. DOX-loaded SNPs demonstrated GSH-responsive drug release in A549 cells, with enhanced fluorescence and cytotoxicity compared to free DOX (Figure 7). In vivo, SNP/DOX showed prolonged circulation and preferential tumor accumulation relative to free DOX. Notably, SNP/DOX exhibited superior antitumor efficacy, achieving a tumor inhibition rate of 97%, compared to 75% for free DOX and 87% for non-cleavable DOX-loaded nanoparticles, along with significant reductions in tumor volume and weight (Figure 7).

In 2018, Guo et al. reported a disulfide-crosslinked polypeptide nanogel based on poly(L-lysine)–poly(L-phenylalanine-co-L-cystine) (PLL–P(LP-co-LC)) as an effective peptide-based platform for delivering 10-hydroxycamptothecin (HCPT) in the treatment of orthotopic bladder cancer [54]. The positively charged PLL–P(LP-co-LC) system significantly increased the retention time and enhanced the tissue permeability of HCPT in the rat bladder wall. Additionally, the reduction-responsive nanogel (NG/HCPT) enables rapid and targeted drug release in bladder cancer cells. As a result, NG/HCPT effectively inhibits the proliferation of human bladder cancer 5637 cells in vitro and demonstrates enhanced antitumor efficacy in an orthotopic rat bladder cancer model in vivo. Das and co-workers reported an ultrasmall peptide-based hydrogel formed via disulfide-linked dimerization of PyKC peptides for targeted DOX delivery [55]. Upon exposure to intracellular glutathione, the system undergoes cleavage, releasing DOX, generating ROS, and inducing apoptosis in triple-negative breast cancer (MDA-MB-231) cells.

Among the reported GSH-responsive therapeutic delivery systems, the doxorubicin-loaded self-adaptive nanoparticle (SNP/DOX) demonstrated remarkable antitumor efficacy. In the tumor microenvironment, the nanoparticles decreased in size from approximately 145 nm to 40 nm and underwent a reversal in surface charge, changing from −7.4 to +8.2 mV. This facilitated improved tumor penetration and cellular uptake. In a lung carcinoma model, SNP/DOX achieved nearly 97% tumor inhibition after 20 days of treatment while maintaining stable body weight, indicating minimal systemic toxicity [53].

Similarly, a cationic polypeptide nanogel loaded with hydroxycamptothecin (HCPT) showed promising therapeutic performance against bladder cancer. Histological analysis revealed that approximately 69.4% of tumor cells underwent necrotic cell death, highlighting its potent anticancer activity [54]. Although these delivery systems employ different targeting and release mechanisms, both exhibited outstanding tumor-suppressive effects and underscore the potential of GSH-responsive peptide-based nanocarriers for effective cancer therapy.

### 3.2. pH-Stimulated Peptide

Acidosis is a hallmark of cancer cells and tumor microenvironments. Typically, the extracellular pH (pHe) in tumors is lower (~6.3–6.7) than in normal tissues, creating a proton gradient that drives proton efflux from the intracellular to the extracellular space. This acidic environment arises primarily from metabolic reprogramming, notably the Warburg effect, in which cancer cells preferentially undergo glycolysis even under aerobic conditions, leading to excessive production of lactic acid instead of complete oxidation to pyruvate.

In addition, the upregulated pentose phosphate pathway contributes to increased CO_2_ generation, which is transported to the cell surface. There, carbonic anhydrase catalyzes the conversion of CO_2_ to carbonic acid, further contributing to extracellular acidification [56]. Collectively, these processes result in a distinctly acidic tumor microenvironment. Exploiting the acidic nature of the tumor microenvironment, numerous pH-responsive drug delivery systems have been developed by incorporating acid-labile linkages such as acetal and imine bonds [57,58,59]. In 2014, Stupp and colleagues developed self-assembling hexapeptide-conjugated amphiphiles containing histidine-rich oligopeptide segments that formed both cylindrical and spherical nanostructured fibers [60]. These nanostructures underwent disassembly under acidic conditions due to protonation of the histidine residues. Both architectures were capable of encapsulating camptothecin; however, the cylindrical nanofibers exhibited approximately 60% loading efficiency and nearly sevenfold higher drug encapsulation capacity compared to the spherical micelles. Furthermore, the cylindrical nanofibers demonstrated improved tumor accumulation, highlighting the influence of nanostructure morphology on drug delivery performance. In 2020, Ge and co-workers developed a peptide hydrogel, IC1-R (CKIKIKIK-IDPPT-KIOIKIKC-NH_2_), with dual pH and GSH responsiveness [61]. The sequence features alternating polar and nonpolar residues, terminal cysteines, and a unique central -IDPPT- motif. Paclitaxel (PTX) was encapsulated with >98% efficiency and released in response to intracellular pH and GSH, inducing significant cytotoxicity in MCF-7 cells. In vivo, the IC1-R–PTX system showed minimal body weight changes, indicating low toxicity, while achieving a tumor inhibition rate of 94.73% after 10 days. In this context, Li and co-workers developed a pH-responsive peptide hydrogel, OE (VKVKVOVK-VDPPT-KVEVKVKV-NH_2_), for co-delivery of gemcitabine and paclitaxel at varying ratios [62]. Drug release studies showed faster release of gemcitabine at acidic pH (5.8), likely due to its higher hydrophilicity. The combination therapy exhibited enhanced anticancer activity against TNBC 4T1 cells in vitro. In vivo, the system enabled tumor-targeted, microenvironment-triggered, and sustained drug release with improved therapeutic efficacy. Chen and co-workers developed an albumin-mimicking nanodrug based on a zwitterionic poly(glutamatyl lysine-co-cysteine) peptide scaffold to enhance pH-triggered tumor targeting by prolonging circulation and promoting cellular uptake [63]. At tumor-like pH 6.7, uptake by MCF-7 cells was markedly faster than Doxil and comparable to free Doxorubicin, whereas uptake by RAW-264.7 cells at physiological pH 7.4 was only ~27% of Doxil. In vivo, the nanodrug showed higher tumor accumulation than in the liver and kidney and achieved significantly improved tumor inhibition (93.2 ± 3.0%) compared to Doxil (54.2 ± 6.5%), along with better maintenance of body weight. In 2024, Wu and co-workers developed a pH-sensitive dual therapeutic hydrogel for retinoblastoma therapy, in which the anti-VEGF fusion protein conbercept and tobramycin were encapsulated within a heptapeptide hydrogel (DDIIIOH-NH_2_) [64]. In vitro, the system showed 68.32% cumulative drug release at pH 6.5. The combination treatment significantly reduced CD31 expression and microvascular density, indicating enhanced inhibition of tumor angiogenesis through sustained drug release. In vivo, the hydrogel resulted in about a 50% reduction in tumor volume after 10 days of treatment.

A wide range of pH-responsive drug delivery systems has been extensively explored and has demonstrated promising therapeutic potential in vitro and in vivo for cancer treatment [65]. These systems are designed to exploit the acidic nature of the tumor microenvironment and intracellular organelles such as endosomes and lysosomes to achieve selective drug release. Despite their advantages, the practical application of pH-mediated drug release remains highly challenging due to the complex, heterogeneous pH landscape within tumors.

One of the major limitations is that the extracellular tumor matrix itself is mildly acidic (pH ~ 6.5–6.8) compared with normal physiological tissues (pH ~ 7.4). This relatively small pH difference can trigger premature destabilization of pH-sensitive nanocarriers, leading to unintended payload leakage before cellular internalization. As a consequence, therapeutic agents may diffuse away from the tumor site, undergo rapid degradation, or induce systemic toxicity, thereby reducing treatment efficacy. Furthermore, tumor acidity is highly heterogeneous and dynamically influenced by hypoxia, metabolic activity, vascularization, and nutrient availability, making precise control of drug release even more difficult.

Additionally, endosomal escape remains one of the key obstacles to efficient intracellular delivery. Following cellular uptake, many peptide-based DDS get trapped within the endosomes and are subsequently trafficked to lysosomes, where the therapeutic cargo may be degraded before reaching its intracellular target. To overcome this limitation, various strategies have been developed, including the incorporation of membrane-disruptive peptides and pH-responsive moieties.

Along these lines, another important complexity arises from the requirement that nanoparticles remain sufficiently stable during blood circulation while retaining the ability to respond rapidly upon cellular uptake. Ideally, the nanocarrier should remain stable and resist premature drug release in the mildly acidic extracellular tumor microenvironment, while allowing efficient activation in the more acidic intracellular compartments, such as endosomes and lysosomes (pH ~ 4.5–5.5). However, this strategy also presents certain limitations, as lysosomes contain various proteolytic enzymes and harsh acidic conditions that may degrade or destabilize some therapeutic agents before they reach their intended intracellular targets. Achieving this balance between extracellular stability and intracellular responsiveness is a major design challenge.

### 3.3. ROS-Triggered Peptide

Reactive oxygen species (ROS) are highly reactive oxygen-containing molecules that readily interact with cellular components. At elevated levels, ROS can damage DNA, RNA, proteins, and the immune defense system, ultimately leading to cell death. Conversely, ROS also play essential roles in cell signaling, including regulation of signaling cascades, apoptosis, and gene expression [66], functioning as both intra- and intercellular messengers.

ROS are mainly produced as byproducts of the mitochondrial electron transport chain and through various metal-catalyzed oxidation processes in biological systems. Partial reduction of oxygen leads to the formation of diverse ROS, such as superoxide (O_2_^−^), hydrogen peroxide (H_2_O_2_), and hydroxyl radicals. To maintain redox balance, cells employ antioxidant defense mechanisms; for example, superoxide dismutase (SOD) catalyzes the conversion of superoxide into H_2_O_2_ and water.

**Figure 8:**
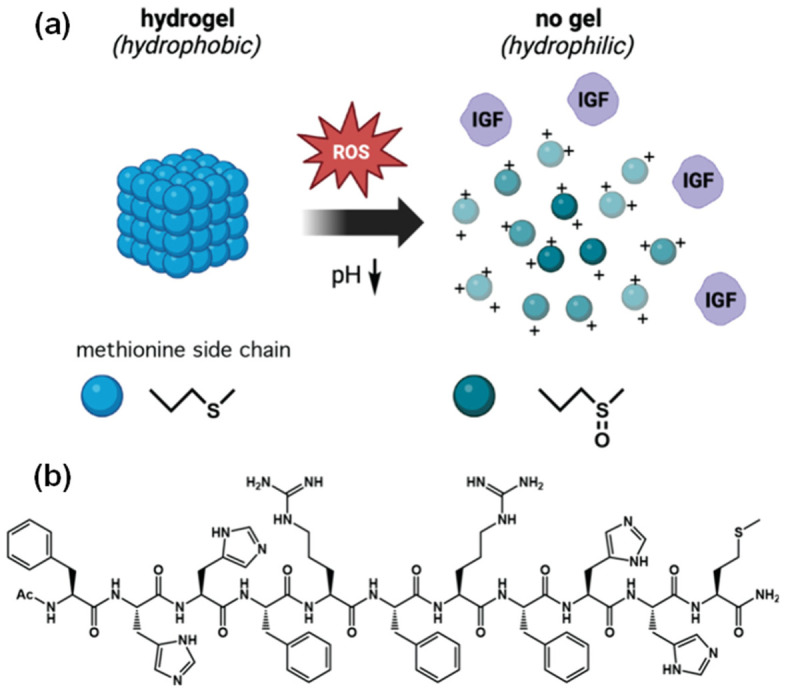
Design of a stimuli-responsive hydrogel for osteoarthritis therapy: (**a**) Schematic illustration of the ROS-scavenging mechanism coupled with targeted delivery of IGF-1 to the diseased site; oxidation of methionine by H_2_O_2_ triggers hydrogel degradation and release of IGF-1 in the inflamed joint. (**b**) Chemical structure of FHHM-11. Figure adapted with permission from [74]. Copyright 2025, American Chemical Society.

In cancer cells, ROS levels are generally elevated compared to normal cells. This distinctive feature has been widely exploited to develop ROS-responsive drug delivery systems for targeted therapeutic applications. Earlier, H_2_O_2_-responsive polymer-based drug delivery systems and peptide-based fluorescent probes were developed for the selective detection and monitoring of ROS, particularly hydrogen peroxide [67,68,69,70]. Zheng and co-workers developed a ROS-responsive peptide-based drug delivery system for hepatic fibrosis, a chronic liver disease [71]. They designed CRGD-modified micelles using an amphiphilic block copolymer, poly(L-methionine-block-Nε-trifluoroacetyl-L-lysine) (PMK), to enable targeted delivery of resveratrol (RES) to activated hepatic stellate cells (aHSCs). These CRGD-PMK micelles selectively accumulated in aHSCs and released RES in response to elevated intracellular ROS levels. In vitro, the system promoted ROS consumption, reduced collagen deposition, and inhibited aHSC activation. In vivo, it effectively alleviated inflammation, suppressed fibrosis progression, and protected hepatocytes in fibrotic mouse models. Muraoka and co-workers developed methionine-containing self-assembling peptides inspired by sulfur-based redox chemistry [72]. These peptides formed hydrogels that underwent a gel-to-sol transition upon oxidation by H_2_O_2_, demonstrating oxidation-responsive behavior. The sensitivity of the hydrogels increased with the number of methionine residues present in the peptide sequence, with the peptide containing three methionine units exhibiting the highest responsiveness and providing effective protection against oxidative cellular damage. The oxidation of methionine residues within peptide or protein chains is strongly influenced by both the concentration of oxidizing agents and the structural accessibility of the methionine side chains [73]. In general, methionine oxidation follows second-order kinetics, highlighting the importance of local microenvironment and reactant availability in determining oxidation efficiency and subsequent structural transitions of the hydrogel system. This system showcases a supramolecular potential candidate against oxidative stress. Makhlynets and co-workers developed a ROS-responsive peptide polymer (FHHM-11) for the treatment of osteoarthritis (OA), a condition associated with elevated ROS levels [74]. The methionine residues in the peptide undergo oxidation to sulfoxide (–S=O), leading to hydrogel degradation and triggered release of the cartilage-repairing growth factor insulin-like growth factor 1 (IGF-1) (Figure 8).

**Figure 9:**
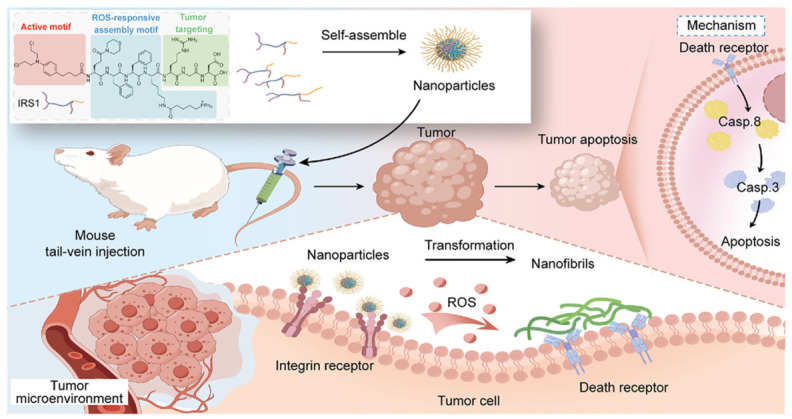
Chemical structure of IRS1 and its selective activation of the extrinsic apoptotic pathway in cancer cells. Figure adapted with permission from [75]. Copyright 2026, American Chemical Society.

In a recent study, Zhang and co-workers developed a small peptide, IRS1, that self-assembles into nanoparticles and preferentially accumulates in uveal melanoma (UM) cells through integrin-mediated targeting [75]. Within the tumor microenvironment, ROS-triggered oxidation of thiomorpholine induces a hydrophobic-to-hydrophilic transition, resulting in nanoparticle-to-nanofibril transformation and exposure of the chlorambucil moiety (Figure 9). This process facilitates covalent interaction with thiol groups of death receptors DR4/DR5 on the cell membrane. IRS1 disrupts membrane integrity and activates the extrinsic apoptosis pathway, inducing cell death in OCM-1A, HeLa, and MDA-MB-231 cells. PCR analysis showed concentration-dependent upregulation of DR4 and DR5, along with increased expression of caspase-8 and caspase-3, confirming apoptosis-mediated cell death. In vivo studies showed that administering IRS1 at a dosage of 10 mg per kg resulted in a significant suppression of tumor growth (Figure 9).

In ROS-mediated peptide delivery systems, oxidation-sensitive amino acids or ROS-cleavable linkers undergo structural disruption or bond cleavage in the presence of ROS such as H_2_O_2_ and peroxynitrite, leading to controlled release of therapeutic agents. These peptide assemblies can self-assemble into nanoparticles, micelles, nanofibers, or hydrogels, improving drug stability, circulation time, and cellular uptake.

In cancer therapy, ROS-responsive peptide systems offer enhanced tumor selectivity, reduced premature drug leakage, minimized systemic toxicity, and improved intracellular drug accumulation. Owing to the intrinsically high ROS levels in cancer cells, these systems can selectively release anticancer agents within tumors while sparing normal tissues. Furthermore, they can be combined with chemotherapeutics or phytochemicals to amplify oxidative stress and induce apoptosis, ferroptosis, or paraptosis-mediated cell death. Beyond cancer therapy, these systems also hold potential for treating stroke, neurodegenerative disorders, cardiovascular inflammation, and osteoarthritis.

### 3.4. Gasotransmitter-Induced Peptide

Gasotransmitters are small gaseous molecules, including carbon monoxide (CO), nitric oxide (NO), and hydrogen sulfide (H_2_S), that function as important signaling mediators in biological systems. They play key roles in regulating immune responses, reducing oxidative stress, and modulating various physiological processes, making them relevant in the treatment of inflammatory diseases.

In cancer, particularly NO and H_2_S exhibit a dual (dose-dependent) role [76,77]. Both are often elevated in tumor cells. At low and sustained concentrations, NO promotes tumor growth, angiogenesis, and cell survival, whereas at higher levels it can induce cytotoxicity, DNA damage, and apoptosis. Similarly, H_2_S is overproduced in cancer cells due to upregulation of enzymes such as cystathionine γ-lyase and cystathionine-β-synthase. At endogenous levels, H_2_S promotes tumor progression by enhancing angiogenesis, cellular metabolism, and chemoresistance.

Given their altered levels in tumors, gasotransmitters can be explored as endogenous stimuli to trigger controlled drug release, enabling precise and effective therapeutic delivery in cancer cells. Yan and co-workers reported an amphiphilic polypeptide containing an o-phenylenediamine-functionalized poly(L-glutamate) segment (PEOPA) that exhibits exceptional sensitivity and selectivity toward nitric oxide (NO) biosignals [78]. These polypeptides can self-assemble into rigid filamentous nanostructures through the synergistic effect of the α-helical secondary structure of the PEOPA blocks and interchain hydrogen bonding among OPA side groups.

At biologically relevant concentrations, NO cleaves the filament-forming OPA motifs, leading to the disassembly of the nanostructures. This NO-responsive behavior enables these polypeptide nanofilaments to function as NO-activated nanocarriers. Such a strategy highlights the potential for NO-mediated drug delivery systems.

Although several nanocarrier-based strategies have been developed to deliver NO to cancer cells, often in combination with chemotherapeutic agents to enhance anticancer efficacy, relatively limited attention has been given to NO-triggered drug delivery systems [79,80]. Further research in this area is essential to enhance therapeutic efficacy and to develop more efficient anticancer drug delivery systems. Matson and co-workers developed self-assembled peptide–hydrogen sulfide donor conjugates (PHDCs), including CA-FEEKS, CA-EEFKS, CA-KSEEF, and CA-KSFEE [81]. These isomeric PHDCs undergo self-assembly in aqueous solution to form diverse nanostructures such as nanoribbons, nanofibers, and nanobelts with varying dimensions. In buffer (pH 7.4), the PHDCs, in the presence of 10 equivalents of the trigger molecule cysteine (Cys)—commonly used in SATO-based materials and mimicking the high intracellular concentration of reduced thiols—facilitate the release of H_2_S in cardiovascular cells. Similar to nitric oxide (NO), several H_2_S-releasing peptide systems have been developed [82,83,84]. However, there remains significant scope for the development of H_2_S-triggered drug delivery systems, as research on H_2_S-responsive drug release is still relatively limited.

There is considerable potential for developing peptide-based drug delivery systems that respond to gasotransmitters, particularly hydrogen sulfide (H_2_S)-triggered nanocarriers, as research in this area is still limited. H_2_S is a significant endogenous signaling molecule involved in processes such as cancer progression, inflammation, angiogenesis, and redox regulation. Elevated levels of H_2_S in various cancers make it an appealing trigger for targeted drug release. Peptide-based systems are particularly promising due to their biocompatibility, biodegradability, structural flexibility, and ability to self-assemble. Despite these advantages, several challenges remain, including variable H_2_S concentrations across tissues, poor stability, premature drug leakage, slow response kinetics, and interference from other biological thiols. Since H_2_S is highly reactive and short-lived, achieving precise drug activation remains difficult. Therefore, future studies should focus on developing highly selective and stable multifunctional peptide nanocarriers. Combining H_2_S responsiveness with other endogenous stimuli such as ROS, pH, or enzymes may further improve targeted drug delivery and therapeutic efficacy.

### 3.5. Enzyme-Stimulated Peptide

Tumor cells exhibit distinctive characteristics, including an acidic microenvironment and a highly heterogeneous milieu. These conditions promote the overexpression of a wide range of enzymes—such as proteases, oxidoreductases, oxidases, and hydrolases—making cancer cells markedly different from normal cells. Common examples include lysosomal proteases (e.g., cathepsins), furin protease, hydrolytic enzymes such as lysophosphatases, alkaline phosphatase, and acid phosphatase, as well as oxidative enzymes like monoamine oxidase and tyrosinase, all of which are frequently overexpressed in cancer cells [85,86,87].

**Figure 10:**
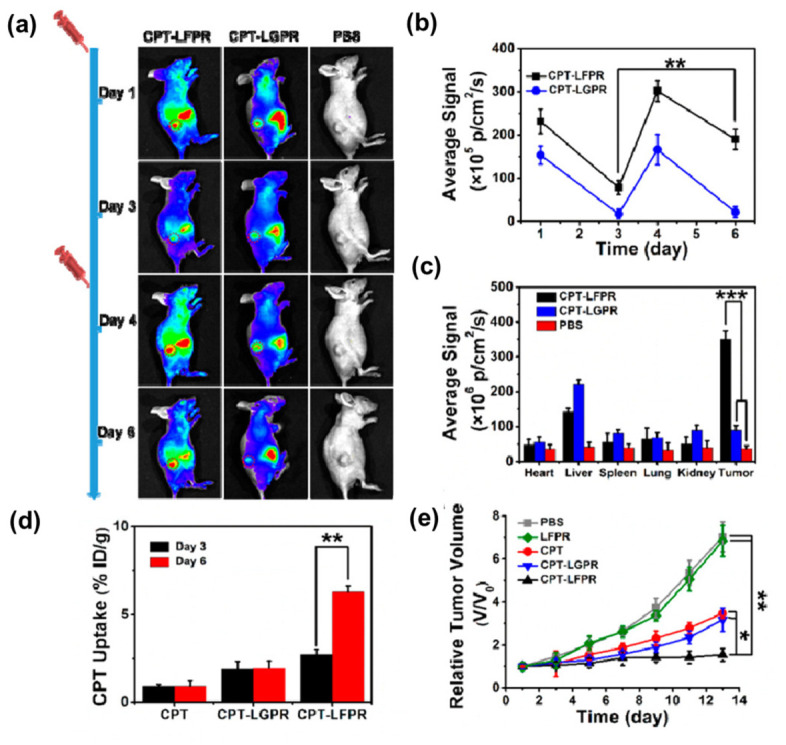
(**a**) In vivo fluorescence images of tumor-bearing mice following i.v. injection of CPT-LFPR, CPT-LGPR, and PBS on days 1 and 4 (tumors indicated by red circles). (**b**) Quantitative fluorescence analysis of tumors at different time points (N = 3). (**c**) Ex vivo fluorescence quantification of tumors and major organs at day 6 (N = 3). (**d**) CPT accumulation in tumors at days 3 and 6, expressed as % injected dose per gram of tissue (% ID/g), after treatment with CPT-LFPR, CPT-LGPR, and CPT (N = 3). (**e**) Tumor volume changes in mice treated with PBS, LFPR, CPT, CPT-LGPR, and CPT-LFPR (N = 5). * *p* < 0.05, ** *p* < 0.01, *** *p* < 0.001. Figure adapted with permission from [93]. Copyright 2019, American Chemical Society.

Considering these enzymes as cancer biomarkers and triggers, various polymer- and nanomaterial-based drug delivery systems have been developed [88,89,90]. In this section, a few representative peptide-based examples of self-cleavable drug delivery systems are presented. Choi and co-workers developed a cathepsin B enzyme-responsive lipid, PEG-GLFG-K(C16)_2_ (PEG-GLFG; polyethylene glycol–Gly-Leu-Phe-Gly-Lys(C16)_2_) [91]. They created liposomes consisting of PEG-GLFG, DOTAP (1,2-dioleoyl-3-trimethylammonium propane, chloride salt), DPPC (dipalmitoylphosphatidylcholine), and cholesterol, which degrade in the presence of the cathepsin B enzyme. Doxorubicin (Dox)-loaded GLFG liposomes (GLFG/Dox) demonstrated significant anticancer activity against HepG2 cells in vitro and effectively inhibited cancer cell proliferation in a zebrafish model. The same group later developed a cathepsin B-responsive amphiphilic peptide delivery system, Arg–His–(Gly-Phe-Leu-Gly)_3_ (RH-(GFLG)_3_). In this design, the arginine moiety enhances cellular uptake, while histidine facilitates lysosomal escape through its buffering capacity [92]. The doxorubicin-loaded nanoformulation exhibited improved anticancer activity in HeLa cells compared to free Dox. Wang’s group developed a peptide material, CPTLVFFGFLG–PEGRGD (CPT-LFPR), that forms nanoparticles under physiological conditions and serves as a scaffold for fibrillar drug systems [93]. After intravenous administration, these nanoparticles accumulate in target tissues via peptide-mediated targeting. In the presence of cathepsin B, cleavage of surface peptides removes the PEG-RGD shell, triggering reassembly into β-sheet nanofibers. This transformation, accelerated by preformed seeds, enhances drug accumulation and retention in tumor cells, enabling in situ formation of sustained-release depots and effective suppression of tumor growth (Figure 10).

Laverty and co-workers developed an injectable hydrogel composed of a tetrabenzylamine–tetraglycine–D-lysine–O-phospho-D-tyrosine peptoid–D-peptide formulation ((NPhe)_4_GGGGk(AZT)y(p)-OH) [94]. The hydrogel undergoes esterase-mediated degradation, leading to the controlled release of the antiretroviral drug zidovudine (AZT). Apart from these, other enzyme-stimulated DDSs have been developed [95,96,97]. Enzyme-mediated peptide-based drug delivery systems (DDS) have emerged as promising smart therapeutic platforms because many pathological tissues, particularly tumors, overexpress specific enzymes such as matrix metalloproteinases, cathepsins, phosphatases, and proteases. These enzymes can selectively cleave peptide sequences or trigger structural transformation of peptide nanocarriers, enabling site-specific drug release with reduced systemic toxicity. Peptide-based DDS offers several advantages, including excellent biocompatibility, biodegradability, facile synthesis, and the ability to self-assemble into nanoparticles, nanofibers, or hydrogels for efficient drug encapsulation and delivery.

Despite these advantages, several challenges remain. Achieving optimal cell-specific uptake, avoiding premature enzymatic degradation during circulation, maintaining carrier stability, and ensuring selective activation within diseased tissues are still difficult. Furthermore, variability in enzyme expression among patients and tumor heterogeneity complicates clinical translation. Therefore, developing technically simple, highly selective, and clinically viable enzyme-responsive peptide systems remains an important future research direction.

### 3.6. Hypoxia Sensitive Peptide

**Figure 11:**
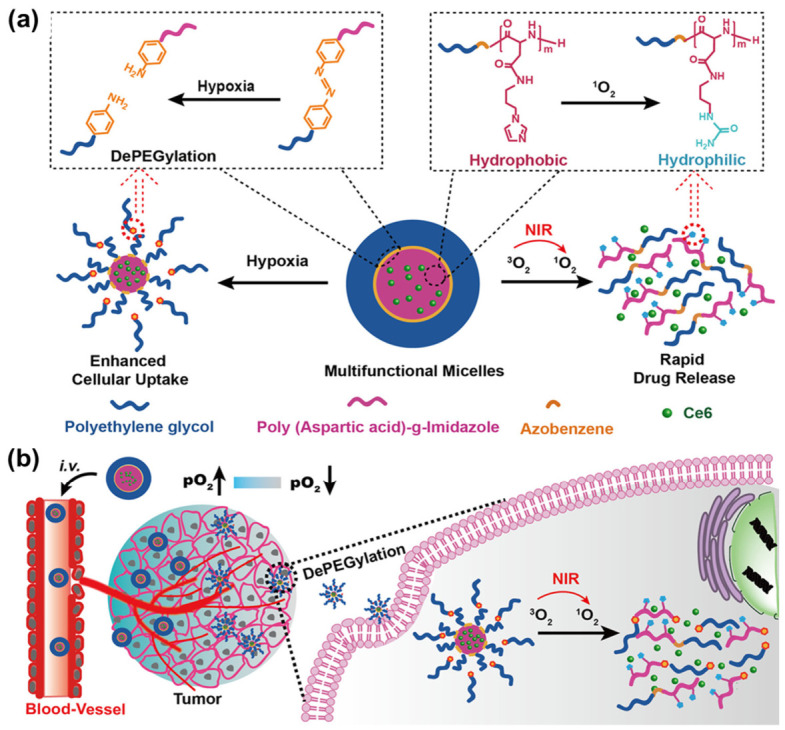
Schematic illustration of hypoxia- and singlet oxygen-responsive tailor-made micelles for enhanced photodynamic anticancer therapy. (**a**) A hypoxia-sensitive azobenzene linker connects the hydrophilic PEG block and hydrophobic polypeptide segment to form self-assembling amphiphilic copolymers loaded with chlorin e6 (Ce6). (**b**) In the tumor microenvironment, hypoxia-induced dePEGylation enhances cellular uptake, while singlet oxygen responsiveness triggers micelle disassembly and rapid Ce6 release, enabling efficient drug delivery and controlled release at the tumor site. Figure adapted with permission from [98]. Copyright 2018, American Chemical Society.

Hypoxia is a critical feature of tumor progression, typically emerging in advanced stages of cancer. It occurs in regions distant from blood capillaries (beyond ~150 μm), where the diffusion of oxygen and nutrients is insufficient. Under these oxygen-deprived conditions, hypoxia-inducible factor-1α (HIF-1α) becomes highly active, promoting multidrug resistance, creating a reducing microenvironment, and contributing to necrotic cell death. Most chemotherapeutic agents fail to achieve the desired efficacy under tumor hypoxia [95]. Interestingly, certain aromatic functionalities, such as nitro and azo bonds, are highly susceptible to reduction, enabling the selective release of chemotherapeutic agents in hypoxic regions [96]. Various hypoxia-sensitive prodrugs and nanodelivery systems have been developed in recent years to address this limitation. In this context, Kulkarni et al. reported lipid nanoparticles (LNs) composed of a hypoxia-responsive lipid and a peptide–lipid conjugate. These LNs effectively penetrate hypoxic regions of tumors [97]. Under low oxygen conditions, the hypoxia-responsive lipid undergoes reduction, leading to membrane destabilization and the release of the encapsulated drug, gemcitabine. In 3D spheroid pancreatic cancer (BxPC-3) cells, this system reduced cell viability by approximately 35% under hypoxic conditions. Zhao and colleagues developed a photodynamic therapy (PDT) system to target hypoxic tumors using an imidazole-conjugated methoxy poly (ethylene glycol)–azobenzene–poly (aspartic acid) copolymer (Figure 11). The system self-assembled into micelles (189 ± 19 nm) and efficiently encapsulated the photosensitizer chlorin e6 (Ce6) with a loading efficiency of 4.1 ± 0.5% (*w*/*w*) [98]. Hypoxia-induced dePEGylation and singlet oxygen-triggered Ce6 release were successfully demonstrated in both aqueous buffer and Lewis lung carcinoma (LLC) cells (Figure 11).

Jiang and colleagues developed a tumor microenvironment-activatable drug delivery system (^TAT+Azo^NPs) to enhance photodynamic therapy (PDT)-induced bio-reductive chemotherapy. The TAT peptide enhances cellular uptake and tumor penetration [99]. These nanoparticles co-deliver chlorin e6 (Ce6) and the hypoxia-activated prodrug tirapazamine (TPZ), accumulating in both proximal and distal tumor regions. The azobenzene linker is stable under normoxia but cleaves in hypoxia, releasing TPZ upon PDT-induced hypoxia. In a 4T1 triple-negative breast tumor model, ^TAT+Azo^NPs@(Ce6+TPZ)L achieved an impressive tumor growth inhibition rate of 86.6%.

Additionally, Zheng et al. developed a hypoxia-responsive self-assembled peptide hydrogel for ischemic stroke therapy [100]. This hydrogel, made from azocalixarene (CA) complexed with CY5-DM and FTY720, allows hypoxia-responsive release. The self-assembled hydrogel provides inflammation suppression and promotes recovery after treatment. Despite the development of various hypoxia-sensitive drug delivery strategies, treating hypoxic tumors remains difficult due to the complex and heterogeneous tumor microenvironment. Hypoxia, a hallmark of many solid tumors, results from abnormal vascularization and insufficient oxygen supply, promoting tumor progression and resistance to therapies. Currently, there are no FDA-approved drugs specifically targeting hypoxic conditions.

A significant challenge in hypoxia-mediated therapies, especially photodynamic therapy (PDT), is the limited oxygen availability in tumor tissues. Both Type I and Type II PDT rely on molecular oxygen to produce reactive oxygen species (ROS), making conventional PDT less effective under hypoxic conditions. Innovative strategies are essential to enhance PDT’s efficacy in oxygen-deficient tumors. These include oxygen self-supplying systems, hypoxia-activated prodrugs, ROS-amplifying nanoplatforms, and combined therapies such as immunotherapy and sonodynamic therapy. These approaches may help overcome hypoxia-related resistance and improve treatment outcomes in solid tumors.

### 3.7. Multi Stimuli DDS

Even though several studies have reported single-stimulus-responsive peptide-based drug delivery systems and considerable efforts have demonstrated their promising in vivo therapeutic performance, relying on only one biological trigger is often insufficient to achieve highly precise and selective cargo release within cancer cells. In the tumor microenvironment, many endogenous stimuli such as acidic pH, elevated glutathione (GSH), reactive oxygen species (ROS), and enzyme overexpression are not unique to cancer tissues and may also be present, to some extent, in normal tissues and physiological compartments. As a result, single stimuli-responsive systems can suffer from premature drug leakage, insufficient targeting accuracy, and reduced therapeutic selectivity.

For example, the extracellular region of tumor tissues is generally more acidic than that of normal tissues due to enhanced glycolytic metabolism and lactic acid production. However, intracellular organelles such as lysosomes and endosomes in both cancerous and normal cells also possess acidic environments. Consequently, pH-responsive nanocarriers may undergo undesired activation and drug release even in healthy cells, thereby limiting their selectivity. Similarly, glutathione (GSH)-responsive systems have attracted significant attention because intracellular GSH concentrations in cancer cells are relatively higher than those in extracellular fluids. Nevertheless, GSH is also abundantly present in blood plasma and normal cells, which makes GSH-mediated delivery systems vulnerable to premature or off-target drug release before reaching the tumor site.

To overcome the intrinsic limitations associated with single-stimulus-responsive delivery systems, dual- or multi-stimuli-responsive peptide-based nanocarriers have emerged as highly promising platforms for cancer therapy. These advanced systems are designed to respond simultaneously or sequentially to multiple tumor-associated triggers, such as pH/GSH, pH/ROS, enzyme/pH, or temperature/redox combinations. The integration of multiple stimuli significantly improves the precision of nanocarrier activation, minimizes premature drug leakage, enhances tumor selectivity, and promotes controlled intracellular drug release. As a result, dual- and multi-stimuli-responsive peptide nanocarriers can achieve superior therapeutic efficacy and reduced side effects compared to conventional single stimuli-responsive delivery systems. In this context, in 2015, the Zhang group developed a dual stimuli-responsive layer-by-layer (LbL) self-assembled platform driven by host–guest interactions between a poly(aspartic acid-graft-β-cyclodextrin) (PASP-g-β-CD) layer and a poly(acrylic acid-graft-azobenzene-graft-proline-leucine-glycine-valine-arginine-adamantane) (PAA-g-Azo-g-PLGVR-AD) layer. In this system, drugs were encapsulated within the hollow central cavity. In contrast, small-molecule drugs modified with α-cyclodextrin (α-CD) were loaded onto the layers through host–guest interactions between α-CD and azobenzene (Azo) [101].

Importantly, the linkers between the host and guest layers could be cleaved by hydrolysis of the PLGVR peptide sequence by matrix metalloproteinases (MMPs), resulting in gradual capsule dissociation and subsequent release of macromolecular drugs. In contrast, UV irradiation triggered the release of α-CD-modified small-molecule drugs through disruption of the α-CD–Azo interaction. Thus, both endogenous stimuli (MMPs) and exogenous stimuli (UV light) independently enabled controlled and even stepwise drug release.

To demonstrate this concept, the authors employed dual fluorophores as drug mimics: FITC was released through MMP-mediated hydrolysis of the PLGVR peptide, whereas rhodamine B was released upon UV irradiation from the α-CD–RhB complex embedded within the porous host–guest LbL nano-self-assembled platform.

**Figure 12:**
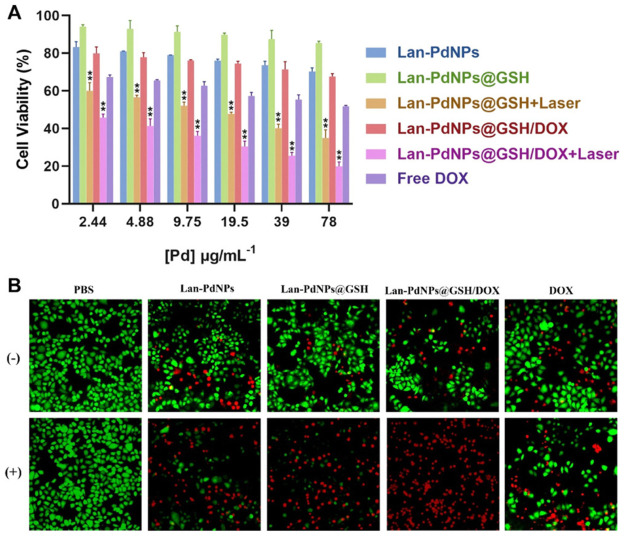
(**A**) Cell viability of HeLa cells treated with different formulations, including Lan-PdNPs, Lan-PdNPs@GSH, Lan-PdNPs@GSH + laser, Lan-PdNPs@GSH/DOX, Lan-PdNPs@GSH/DOX + laser, and free DOX (** *p* < 0.01 vs. free DOX). (**B**) Fluorescence images of treated HeLa cells stained with FDA/PI, showing live (green) and dead (red) cells. Figure adapted with permission from [102]. Copyright 2019, American Chemical Society.

Later, Zhang et al. reported that lanreotide, an octa-amino acid cyclic peptide with the sequence D-Nal-cyclo(Cys-Tyr-D-Trp-Lys-Val-Cys)-Thr-NH_2_, an analog of somatostatin, could act as an internal template for the preparation of Pd-conjugated nanoparticles (Lan-PdNPs). Subsequently, glutathione (GSH) and doxorubicin (DOX) were loaded onto the surface of Lan-PdNPs to construct the nanosystem Lan-PdNPs@GSH/DOX, achieving loading efficiencies of 65.04% and 70.3% for GSH and DOX, respectively [102]. Under near-infrared (NIR) laser irradiation, the nanoparticles efficiently converted light energy into heat, thereby accelerating drug release. In addition, the system exhibited pH-responsive drug release under acidic conditions. The combined effects of light irradiation and acidic pH produced a synergistic enhancement of the antitumor activity of Lan-PdNPs@GSH/DOX in vitro. Notably, Lan-PdNPs@GSH/DOX under light irradiation exhibited the highest antiproliferative activity compared to other nanoparticle formulations, including Lan-PdNPs@GSH and Lan-PdNPs, both in the presence and absence of light irradiation (Figure 12A). Furthermore, live/dead cell staining assays demonstrated that Lan-PdNPs@GSH/DOX induced the greatest extent of cell death, as evidenced by the strongest red fluorescence signal from ethidium bromide-stained dead cells (Figure 12B).

In vivo, laser irradiation increased the photothermal temperature of the system to approximately 60 °C and resulted in a 90.33% reduction in tumor size. Importantly, treated mice did not exhibit significant body weight loss, indicating minimal systemic cytotoxicity. Additionally, histopathological analysis using H&E staining revealed no significant inflammation or damage in major organs.

Then, in 2021, the Liu group developed crosslinked methoxy poly(ethylene glycol)-g-poly(aspartic acid)-g-tyrosine (CPPT) nanoparticles containing a disulfide-crosslinked interlayer and a calcium phosphate (CaP) shell. These pH- and redox-dual-sensitive polypeptide-based organic–inorganic hybrid nanoparticles encapsulated curcumin (Cur) within the hydrophobic micellar core, while doxorubicin hydrochloride (DOX) was loaded onto the hydrophilic segment of the micelles as well as within the CaP shell [103].

The resulting spherical Cur- and DOX-loaded nanoparticles (CPPT@CaP-CD) effectively minimized premature drug leakage under physiological pH conditions due to their enhanced structural integrity. However, under acidic and hypoxic microenvironments, rapid dissolution of the CaP shell along with cleavage of the disulfide-crosslinked network promoted the release of both drugs, enabling stimuli-responsive and controllable drug delivery. At lower pH, approximately 86.5% of Cur and 92.7% of DOX were released.

**Figure 13:**
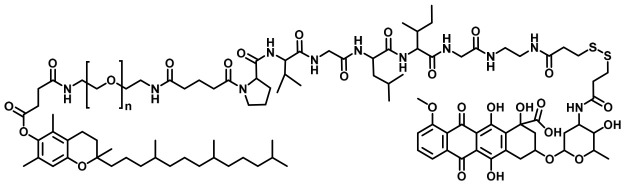
Structure of dual stimuli, MMMP-2 and GSH, responsive peptide. Figure adapted with permission from [104]. Copyright 2023, American Chemical Society.

In vitro studies demonstrated that the co-loaded nanoparticles exhibited significantly higher cytotoxicity against A549 cells compared to the free-drug combination. Furthermore, fluorescence imaging analysis revealed enhanced cellular uptake and greater intracellular release of both DOX and Cur, particularly within the nucleus. Chen et al. reported a dual stimuli-responsive peptide-based micellar system, TPGS3350-PVGLIG-SS-DOX (TPGS3350-PVGLIG-DOX/TPSD), designed to reduce off-target cytotoxicity while improving therapeutic efficacy (Figure 13) [104]. The nanosystem incorporated an extracellular matrix metalloproteinase-2/9 (MMP-2/9)-responsive PVGLIG peptide sequence along with an intracellular glutathione (GSH)-responsive disulfide linkage, enabling sequential dual-stimuli-triggered drug release.

The TPSD micelles loaded with doxorubicin (TPSD-D) were cleaved by MMP-2, exposing the RGD peptide and enhancing cellular uptake in tumor cells. This system demonstrated a significant anticancer effect on triple-negative breast cancer 4T1 cells, with an IC50 value of 2.08 μg/mL.

In vivo studies with 4T1 tumor-bearing mice indicated that TPSD-D significantly reduced tumor volume and weight compared to free doxorubicin (DOX), while causing minimal body weight loss and reduced systemic toxicity. Histopathological analyses revealed specific morphological changes in tumor tissues, such as tissue wrinkling and vacuole formation, with no significant damage to other major organs. In the current year, Wang and co-workers developed a peptide-based nanocarrier composed of arginine-rich terminal residues that functioned as nucleic acid-binding units, along with a serine-rich peptide backbone containing targeting and anti-adhesion sequence modules [105]. The system incorporated lysosomal escape motifs, including RRGK and leucine-repeat sequences, as well as tumor-targeting ligands such as RGDK and GYQTI. This multifunctional peptide nanocarrier demonstrated efficient delivery of both siRNA and mRNA in vitro and in vivo through MMP-7-induced cleavage of the serine-rich unit.

Overall, dual- or multi-stimuli-responsive drug delivery systems, involving combinations of endogenous stimuli or the integration of exogenous and endogenous stimuli, have demonstrated highly promising therapeutic outcomes in both in vitro and in vivo studies. These advanced platforms offer improved site-specific drug release, enhanced cellular uptake, reduced premature leakage, minimized systemic toxicity, and superior antitumor efficacy compared to conventional delivery systems. In particular, the synergistic interplay between multiple stimuli enables more precise spatiotemporal control over drug release within the complex tumor microenvironment.

As summarized in Table 3, endogenous stimuli-responsive peptide-based drug delivery systems have gained significant attention due to their effective pharmacokinetic properties and, in several cases, their minimal toxicity toward normal tissues and major organs.

## 4. Discussion

The above-mentioned peptide-based stimuli-responsive drug delivery systems highlight the current landscape of peptide platforms and their versatility in drug delivery. As essential biological components, peptides function not only as carriers but also serve as targeting agents—for example, cyclic RGD motifs for cancer targeting and PSA (prostate-specific antigen) as a prostate cancer biomarker.

It is worth highlighting that amongst peptide-based drug delivery systems, peptide–drug conjugates (PDCs) represent a unique paradigm by combining the targeting capabilities of peptides along with the activity of the payloads in chemically defined constructs. PDCs are often easier and straightforward to construct compared to nanoparticle-based systems, offering also high reproducibility, and ease of characterization. However, a key disadvantage is their lower drug-loading capacity, low stability, rapid clearance, and often suffer from poor intracellular delivery. Therefore, PDCs represent a complementary strategy within the broader peptide-based drug delivery portfolio.

Within the context of drug delivery systems (DDS), recent examples of peptide-based platforms span both exogenous and endogenous stimulus-responsive systems. These systems incorporate a wide range of warheads, linkers, and peptide sequences to construct targeted drug delivery architectures that minimize off-target effects and reduce toxicity to surrounding healthy cells and tissues. Notably, magnetic fields were first employed as external stimuli for controlled cargo release around 1980 [10]. Subsequently, Kim and co-workers demonstrated temperature-triggered release of indomethacin from poly(NIPAAm-co-BMA) [13], marking significant progress in exogenous stimuli-responsive drug delivery systems.

Further advancing this field, Kim’s group utilized elastin-like polypeptides (ELPs) to develop doxorubicin-loaded liposomes functionalized with an αvβ3 integrin-binding peptide, achieving approximately fivefold higher tumor-specific accumulation [18]. In addition to doxorubicin, thermosensitive peptide-based delivery systems have been explored for drugs such as paclitaxel (PTX) [19].

Moreover, dual stimuli-responsive systems, such as temperature- and pH-sensitive gelatin/peptide composite hydrogels (Gel/PEP), have been developed for the controlled delivery of agents like chelerythrine [22], leveraging both endogenous and exogenous stimuli to improve clinical outcomes. Photo-caging strategies have also been adopted for targeted delivery, wherein nanocages incorporate light-cleavable linkers that are activated by NIR laser, enabling controlled release of chemotherapeutics through structural deformation of the carrier peptide [26,27,28].

Furthermore, ultrasound-responsive peptide hydrogel strategies have emerged as mild, non-invasive external stimuli for drug delivery. These systems not only enable chemotherapeutic delivery but also generate reactive oxygen species (ROS) in a controlled manner [33], thereby disrupting tumor progression through mitochondrial dysfunction. In addition, magnetic field-responsive peptide-based drug delivery systems have gained considerable attention. These systems enable controlled drug release while simultaneously generating localized hyperthermia under an alternating magnetic field, thereby offering a dual therapeutic effect for effective cancer treatment [42].

Overall, exogenous stimulus-responsive drug delivery systems have advanced significantly, particularly with MRI- and ultrasound-guided delivery approaches, whose effectiveness has been demonstrated in clinical settings [106,107]. Ongoing efforts continue to focus on enhancing their performance and broadening their applications.

Similarly, endogenous stimuli-responsive peptide-based drug delivery systems are well established. Glutathione (GSH), a key redox regulator, is often overexpressed in cancer cells, and this feature has been widely exploited for targeted drug delivery. Various peptide-based hydrogels and self-assembling systems have been developed to deliver drugs such as doxorubicin (DOX), paclitaxel (taxol), and HCPT, demonstrating promising results in cancers including hepatocellular carcinoma and triple-negative breast cancer [50,51,52]. Among pH-responsive drug delivery systems, a dual pH/GSH-responsive peptide nanocarrier loaded with paclitaxel achieved 94.73% drug release and demonstrated remarkable antitumor efficacy, resulting in approximately 94.73% tumor volume reduction in an in vivo breast cancer model [61]. Similarly, a combination therapy based on gemcitabine and paclitaxel showed promising therapeutic outcomes in both in vitro and in vivo studies [62]. In another study, a zwitterionic poly(glutamyl-lysine-co-cysteine) peptide scaffold was developed to enhance pH-triggered doxorubicin delivery, achieving about 93.2% tumor volume reduction. Overall, these findings suggest that dual stimuli-responsive delivery systems generally offer superior therapeutic performance, while combination chemotherapy represents an attractive alternative strategy for improving anticancer efficacy. ROS-mediated peptide disintegration has emerged as a promising strategy for drug release. In particular, peptide residues containing methionine or thiomorpholine units undergo oxidation (S → S=O), leading to structural deformation and subsequent release of anticancer drugs [72,73]. Additionally, the self-assembling peptide molecule IRS1, which releases chlorambucil through ROS-triggered oxidation, demonstrated significant antitumor activity in a uveal melanoma (UM) tumor model. After 15 days of treatment, the tumor volume increased by only about 4.1-fold in the IRS1-treated group, whereas the untreated control tumors exhibited approximately a 7-fold increase in volume, highlighting the therapeutic efficacy of this ROS-responsive peptide system [73]. Despite extensive research on gasotransmitter-based chemotherapeutics, including photodynamic therapy (PDT) [108,109,110,111], this strategy has been only limitedly explored in peptide-based drug delivery systems. Therefore, there is considerable scope to further exploit this approach.

In the context of enzyme-responsive drug delivery systems, Choi and co-workers developed a self-assembling polymeric platform for doxorubicin delivery in which the cathepsin B-sensitive peptide sequence was cleaved at the lysine residue of the C-terminal dipeptide, enabling controlled drug release [91]. The same group also reported doxorubicin-loaded liposomes containing a Gly-Leu-Phe-Gly (GLFG) linker, where arginine residues enhanced cellular uptake, while histidine residues promoted lysosomal escape through a protonation–deprotonation buffering effect [92]. This lysosomal escape mechanism is particularly important for the safe transport of therapeutic agents from lysosomes and endosomes, which are rich in proteolytic enzymes that may otherwise degrade or chemically modify the drug, thereby reducing its therapeutic efficacy. Wang and co-workers developed a camptothecin (CPT)-loaded peptide delivery system, CPTLVFFGFLG–PEGRGD (CPT-LFPR), which demonstrated remarkable in vivo performance [93]. In a HeLa tumor-bearing mouse model, CPT-LFPR achieved approximately 2.5-fold higher tumor accumulation than free CPT after 6 days and exhibited significantly enhanced antitumor efficacy. While Wang’s study established the in vivo therapeutic potential of peptide-based enzyme-responsive delivery systems, Choi and colleagues highlighted the importance of histidine residues in promoting lysosomal escape through a proton-buffering mechanism, thereby facilitating the safe intracellular transport of therapeutic payloads [94]. Collectively, these findings suggest that incorporation of histidine residues into peptide conjugates may be an effective strategy for improving endosomal/lysosomal escape and enhancing drug efficacy. Nevertheless, comprehensive pharmacokinetic, pharmacodynamic, biodistribution, and long-term safety studies are still required before these systems can advance toward clinical translation.

In view of hypoxia therapy, Kulkarni et al. developed a hypoxia-responsive gemcitabine delivery system that reduced the viability of 3D pancreatic cancer spheroids (BxPC-3) by approximately 35% under hypoxic conditions [97]. Zhao and co-workers reported an azoreductase-responsive self-assembled peptide system encapsulating chlorin e6 (Ce6), achieving a Ce6 loading efficiency of 4.1 ± 0.5% and exhibiting effective ROS-mediated anticancer activity against Lewis lung carcinoma. In vivo, the treated tumors were approximately 3-fold smaller than those in the untreated group after 16 days, while H&E staining indicated substantial recovery of normal tissue architecture [98]. Similarly, Jiang and colleagues developed ^TAT+Azo^NPs@(Ce6+TPZ)L, a hypoxia-responsive platform that combines photodynamic therapy (PDT) with chemotherapy and achieved an impressive TNBC tumor inhibition rate of 86.6%. Since oxygen deficiency in hypoxic tumors inherently limits PDT efficacy, these findings suggest that combining PDT with chemotherapy represents a more effective therapeutic strategy, as demonstrated by Jiang and co-workers [99]. However, there remains a significant gap in the clinical translation of nanodrug delivery systems capable of achieving effective therapeutic outcomes against tumor hypoxia.

In the field of multi-stimuli-responsive drug delivery, several peptide-based systems have been reported. Early studies demonstrated matrix metalloproteinase (MMP)- or UV-triggered release of model fluorophores such as FITC and rhodamine, primarily as proof-of-concept systems rather than therapeutic platforms [101]. In contrast, Zhang and co-workers developed Lan-PdNPs@GSH/DOX, a dual-responsive nanoplatform that released therapeutic cargo in response to acidic pH and photoirradiation, achieving 90.33% tumor inhibition in vivo with minimal toxicity to normal organs [102]. Similarly, a peptide-based nanocarrier co-loaded with curcumin and doxorubicin exhibited efficient pH- and redox-triggered drug release under tumor-relevant endogenous stimuli in vitro [103]. Another notable system, TPGS3350-PVGLIG-SS-DOX, responded to both MMP-2/9 and GSH, resulting in effective DOX release and significant anticancer activity in a TNBC model [104].

Despite their promising therapeutic performance, most of these systems did not explicitly address endosomal/lysosomal escape, a critical barrier for efficient intracellular drug delivery. In this regard, an elegant peptide delivery platform incorporating lysosomal escape-promoting motifs, including RRGK and leucine-repeat sequences, enabled efficient intracellular delivery of both siRNA and mRNA through MMP-7-induced cleavage of a serine-rich linker, demonstrating efficacy in both in vitro and in vivo models [105]. Such lysosomal escape strategies are particularly important for nucleic acid- and peptide-based therapeutics, where endosomal entrapment can severely limit biological activity. Multi stimuli-responsive systems have demonstrated excellent pharmacokinetic properties, tumor-specific localization, and minimal toxicity to normal tissues. However, further studies are required to collect more comprehensive data before progressing to clinical trials. A systematic evaluation is still needed to achieve more conclusive and fruitful outcomes.

Despite these encouraging developments, several challenges still need to be addressed before these systems can achieve successful clinical translation. Critical factors such as large-scale reproducibility, the appropriate selection and combination of stimuli, optimization of stimulus sensitivity, long-term biocompatibility, biodegradability, and pharmacokinetic stability require further investigation.

From a scalability perspective, large scale peptide synthesis, purification, conjugation, and formulation must all be reproducible, cost-effective, and compatible with GMP manufacturing guidelines. Regulatory approval also requires a fully characterized product, including peptide purity, consistency amongst different batches, stability and degradation products where applicable.

In addition, the pharmacokinetic/pharmacodynamic behavior must be optimized, since peptides can be unstable due to rapid degradation by proteases or clearence by the kidneys. Strategies such as cyclization, stapling, D-amino acid incorporation, PEGylation, lipidation, or nanoparticle encapsulation can improve blood circulation time and biodistribution. However, these modifications need to be carefully executed since they might alter the safety and efficacy profiles. Additionally, although peptides are often considered highly biocompatible, immunogenicity remains a key translational concern, especially for peptide-decorated nanomaterials. Therefore, peptide-based DDS should be extensively evaluated through cytokine release (the immune system can activate immune cells leading to the release of inflammatory cytokines), complement protein activation (i.e., PEGylated liposomes can induce Complement Activation-Related PseudoAllergy), and long-term toxicity studies (repeated administration could lead to elevated toxicity).

Moreover, differences in the tumor microenvironment across various cancer types may affect the responsiveness and therapeutic effectiveness of these systems, highlighting the need for more thorough optimization and standardization.

However, although many dual- and multi-responsive nanosystems (i.e., combination of pH-, enzyme-, redox-, ROS- and temperature-responsive systems) have shown excellent preclinical performance, only limited clinical data are currently available. In conclusion, extensive vivo validation, toxicity assessment, and well-designed clinical studies are still required to establish their safety, efficacy, and translational potential for eventual human therapeutic applications.

## 5. Conclusions and Future Perspectives

Peptides represent a unique class of naturally derived, biocompatible polymeric materials with broad applications in biomedical science, particularly in chemotherapy. They can function as therapeutic agents, targeting ligands for selective tumor recognition, and stimuli-responsive carriers for controlled drug delivery. Owing to these intrinsic advantages, stimuli-responsive peptide systems have emerged as promising platforms for precise and site-specific anticancer therapy. By responding to tumor-associated triggers such as pH, redox gradients, enzymatic activity, hypoxia, and reactive oxygen species (ROS), these systems enable spatiotemporal control of drug release, thereby enhancing therapeutic efficacy while minimizing systemic toxicity.

Additionally, exogenous stimuli such as thermal triggers, light (photo-irradiation), magnetic fields, and ultrasound are highly aligned with practical applications in chemotherapeutic drug delivery systems. These externally applied triggers allow precise spatial and temporal control over drug release, enabling activation specifically at the tumor site while minimizing damage to healthy tissues. For instance, phototriggered systems can use near-infrared light for better tissue penetration and controlled activation. Additionally, thermal and magnetic stimuli can enhance drug accumulation and release by promoting localized heating or using magnetic guidance. Ultrasound, on the other hand, offers a non-invasive approach to improve tissue permeability and trigger drug release via acoustic cavitation. Altogether, these exogenous strategies significantly enhance the efficacy, selectivity, and safety of targeted chemotherapy.

Despite significant progress, several challenges must be addressed before widespread clinical translation can be realized. These include improving in vivo stability against enzymatic degradation, ensuring efficient tumor penetration and accumulation, minimizing off-target effects, and achieving scalable and cost-effective synthesis. Additionally, the complexity of the tumor microenvironment and interpatient variability necessitate more robust and adaptable delivery systems. Another key barrier is achieving efficient endosomal escape while minimizing cytotoxicity, and therefore, further optimization is required to enhance the intracellular bioavailability and therapeutic efficacy.

Looking forward, the integration of multifunctional peptide platforms with nanotechnology, artificial intelligence-guided design, and precision medicine approaches holds great promise. Advanced systems capable of dual- or multi-stimuli responsiveness, real-time imaging (theranostics), and combination therapies (e.g., chemo–photodynamic–immunotherapy) are expected to redefine targeted cancer treatment. Furthermore, the development of personalized peptide-based delivery systems tailored to specific tumor profiles could significantly improve clinical outcomes.

In conclusion, stimuli-responsive peptides represent a versatile and transformative strategy for targeted anticancer drug delivery. Continued interdisciplinary research and technological innovation will be crucial to overcoming current limitations and unlocking their full potential in next-generation cancer therapeutics.

## Figures and Tables

**Table 1:** Physicochemical properties of Lp–peptide hybrids: Hydrodynamic size, polydispersity, zeta potential, and phase transition temperature (Tm) of Lp–peptide, Lp–CHOL, and LTSL. *^a^*. Table adapted with permission from [17]. Copyright 2012, American Chemical Society.

Liposome Composition	Lipid/Peptide (mol/mol)	Hydrodynamic Diameter (nm)	Polydispersity Index (PDI)	Zeta-Potential (mV)	Phase Transition *T*_m_ (^o^C)
DPPC/DSPC/DSPE-PEG_2000_ (90:10:5)					
Lp		123 ± 11.0	0.10 ± 0.050	−12.0 ± 3.00	42.59
Lp-peptide 600:1	600:1	128 ± 0.40	0.07 ± 0.003	−8.00 ± 0.82	42.63
Lp-peptide 200:1	200:1	114 ± 1.70	0.06 ± 0.020	−9.89 ± 1.32	42.95
Lp-peptide 100:1	100:1	128 ± 1.62	0.05 ± 0.003	−9.10 ± 0.45	42.65
DPPC/DSPC/DSPE-PEG_2000_/CHOL (90:10:5:0.5) (Lp-CHOL 200:1)		126 ± 18.0	0.09 ± 0.055	−13.3 ± 0.51	42.77
DPPC/MSPC/DSPE-PEG_2000_ (90:10:4) (LTSL)		97.0 ± 5.01	0.07 ± 0.03	−9.69 ± 0.11	41.39

*^a^* The results are expressed as mean ± STD, *n* = 3. DPPC, 1,2-dipalmitoyl-sn-glycero-3-phosphocholine; DSPC, 1,2-distearoyl-sn-glycero-3-phosphocholine; MSPC, 1-stearoyl-2-hydroxy-sn-glycero-3-phosphocholine; DSPE-PEG_2000_, 1,2-distearoyl-sn-glycero-3-phosphoethanolamine-N-[methoxy(polyethylene glycol)-2000; CHOL, cholesterol.

**Table 2:** Representative exogenous stimuli-responsive peptide polymer-supported drug delivery systems and their therapeutic outcomes.

Peptide	Drug	Stimulator	Application	Outcome	Ref.
[VSSLESKVSSLESKVSKLESKKSKLESKVSKLESKVSSLESK]-NH2	DOX	Temp.	Both in vitro and in vivo studies were performed.	3-fold higher tumor specific accumulation	[17]
poly(g-ethyl-L-glutamate)-poly(ethylene glycol)-poly(g-ethyl-L-glutamate)	PTX	Temp	Both in vitro and in vivo studies were conducted using HCC tumor models.	Tumor volume reduces, no cytotoxicity to normal tissue	[18]
DSPE-PEG-cRGD:cholesterol:stearoyl-ELP	DOX	Temp	Both in vitro and in vivo studies were performed.	In vivo 5-fold tumor (MCF7) specific accumulation	[19]
P21-ELP1-Bac: gemcitabine	Gemcitabine	Temp	Both in vitro and in vivo studies were conducted using pancreatic tumor models.	Showed S1 phase arrest, in both in vitro and in vivo studies, reduced gem doses	[20]
Ac-FALNLAKD-NH2 (PEP)	Chelerythrine (Che)	pH/Tem.	In vitro studies were performed.	In vitro at ≥37 °C higher release rate of Che	[21]
DSPE-PEG_2000_- CGRRMKWKK	Navelbine	Light	Both in vitro and in vivo studies were conducted using MCF-7 cells.	Under light IC_50_ 2.43, Tumor specific localization, tumor suppression and off target toxicity lower than Navelbine	[22]
DEAdcCE	Ruthenocene	Light	In vitro solution studies were performed.	Real system not used	[23]
PEO_114_-*b*-P(LGA_0.62_-co-COU_0.38_)_34_	Rifampicin	Light	In vitro solution studies were performed.	Application to live cells was not performed	[24]
Nvoc-FF	Insulin-FITC,	Light	In tissue studies.	Photo triggered insulin release	[25]
PDMNBLG-r-PVBLG-8	DNA	Light	Cervical cancer model.	DNA transfected to HeLa cells	[26]
KIIIIKNNCCY	DOX	Light	Both in vitro studies using TNBC 4T1 cells and in vivo evaluations were performed.	Both DOX and hyperthermia reduced tumor volume from 5.5 to 0.2 unit	[27]
PTX-tHSA-NPs-MBs	PTX	HIFU	Both in vitro and in vivo studies were conducted using A549 tumor cells.	High cellular uptake, tumor volume reduces 731 mm^3^ to 133 mm^3^	[31]
Methoxy poly(ethylene glycol)-block-poly(L-alanine-co-glycineco- L-isoleucine) (mPEG-b-P(A-G-I))	Naproxen	Ultrasound/Proteinase K	In PBS buffer solution.	3–6 days required the release of naproxen upon enzyme treatment.	[32]
GenPLPF	Epirubicin	Ultrasound	Both in vitro and in vivo studies were performed.	High deep tissue penetration, tumor volume reduced from 183.67 mm^3^ to 26.54 mm^3^	[33]
BP-CFFFVLKLAKLAKDEVDAKRGARSTA	ROS formation	Ultrasound	Both in vitro and in vivo evaluations were carried out.	It has 70 μm tumor tissue penetration; In metastasis lung cancer nodules reduce to 14.6 ± 5.2 from the control, 159.0 ± 41.1. Mitochondria dysfunction and ROS formation are leading cause of anticancer.	[34]
TCPPGG^D^F^D^F^D^YCG^D^K^D^R^D^K	ROS formation	Ultrasound	In vitro and in vivo in cervical cancer	The coupling-induced assembly strategy triggered mitochondrial dysfunction and reactive oxygen species (ROS) generation, leading to apoptosis. This approach demonstrated a tumor inhibition rate of 73.1%.	[35]
GGGGYSAYPDSVPMMSK	-	Magnetic field	Ovarian cancer model.	Successfully removed metastatic cancer cells from the abdominal cavity.	[40]
TAT-peptide	Fe_3_O_4_ as ROS source	Magnetic field	In vitro studies were conducted using A549 and H358 cells.	Mito membrane depolarization, enhanced lysosomal membrane permeability, Caspase 3/7 expression	[41]
Boc− Phe−Phe−Gly−Gly−COOH	Daunorubicin and Hyperthermia	Magnetic field	In vitro studies were conducted pancreatic carcinoma cells (PANC-1)	About 33% PANC-1 only survived	[42]
GHGVY GHGVY GHGPY GHGPY GHGLYW	Hyperthermia and DOX	Magnetic field	In vitro HepG2 cells	After two cycle 93.1% cell death	[43]
Fmoc−arginine−glycine− aspartic acid	-	-	In vitro cell	Injectable nontoxic hydrogel. Can be used for drug delivery	[44]
SPIO-M2pep	Hyperthermia	Magnetic field	In vitro and in vivo as well	M2 macrophage expression and antitumor cytokines secretion	[45]
THP (KDEPQRRSARLSAKPAPPKPEPKPKKAPAKK)	Hyperthermia	Magnetic field	In vitro human glioblastoma cell	Cell death via ferroptosis and hyperthermia	[46]

**Table 3:** Representative endogenous stimuli-responsive peptide polymer-supported drug delivery systems and their therapeutic outcomes.

Peptide	Drug	Stimulator	Application	Outcome	Ref.
mPEG113-*b*-PLL25/DMMA	DOX	GSH	Both in vitro and in vivo studies were conducted using A549 cells.	The system exhibited prolonged blood circulation with a half-life (t_1/2_) of 20.9 h and achieved a tumor inhibition rate of 75% in an A549 lung tumor model.	[53]
poly(l-lysine)–poly(l-phenylalanine-*co*-l-cystine) (PLL–P(LP-*co*-LC))	10-hydroxycamptothecin (HCPT)	GSH	Both in vitro and in vivo studies were conducted using 5637 bladder cancer cells.	IC_50_ of 3.1 μg mL^−1^ lower than free HCPT; in vivo Ki-67 decreased and caspase3 expression increased. Tumor volume large reduced than the HCPT treated only	[54]
PyKC-dimer	DOX	GSH	In vitro studies were performed using TNBC models, including 4T1 and MDA-MB-231 cells.	DNA fragmented, ROS produce and mitochondrial membrane potential disrupted	[55]
PA OEGK2A6K(C12)	CPT	pH	Both in vitro and in vivo studies were conducted using MDA-MB231 cells	At pH6 showed lowest cell viability compared to the pH 7	[60]
IC1-R(CKIKIKIK-IDPPT-KIOIKIKC-NH2)	PTX	pH/GSH	Both in vitro and in vivo studies were performed.	The system exhibited an IC50 value of 8.468 μg/mL against MCF-7 cells and achieved a tumor volume inhibition rate of 79.23% after 10 days of treatment.	[61]
VKVKVOVK-V^D^PPT-KVEVKVKV-NH_2_	Gem and PTX	pH	Both in vitro and in vivo studies were conducted using 4T1 cells.	The peptide itself was non-toxic; however, the gemcitabine (Gem)- and paclitaxel (PTX)-loaded hydrogel exhibited significant cytotoxicity against 4T1 cells. Furthermore, in vivo studies demonstrated that, after 7 days of treatment, the tumor volume was reduced by 90.84%.	[62]
Poly(glutamatyl lysine-co-cysteine)	DOX	pH	Both in vitro and in vivo studies were performed using MCF-7 cells.	In vivo studies demonstrated a half-life (t_1/2_) of 7.0 h following zero-order release kinetics. After 4 days of treatment, the tumor volume was reduced by approximately 40%, reaching 62.3 ± 24.3 mm^3^.	[63]
DDIIIOH-NH_2_	DOX, conbercept	pH	Both in vitro and in vivo retinoblastoma studies were performed.	The system inhibited Y79 tumor cell growth and angiogenesis in retinal endothelial cells, and also suppressed tumor growth and angiogenesis in vivo.	[64]
ES/CRG-PMK-MCs	Resveratrol	H_2_O_2_	Liver fibrosis	In vivo studies demonstrated higher plasma concentration and a prolonged half-life (t_1/2_) compared to free resveratrol.	[71]
Ac-RMDARMRADMR-NH2	Antioxidant activity	H_2_O_2_	Jurkat cells	The peptide hydrogel exhibited significant antioxidant activity and provided effective detoxification against ROS-induced oxidative stress.	[72]
Ac-FHHFRFRFHHFCONH2	Insulin like growth factor 1, IGF-1)	H_2_O_2_	Chondrocytes and RAW macrophages	The system promoted IGF-1 release while simultaneously reducing ROS generation.	[74]
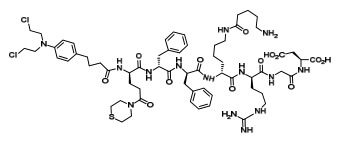	Chlorambucil	H_2_O_2_	MDA-MB231, HeLa cell in vitro and	Tumor weight was significantly reduced from 0.41 g to 0.07 g. Furthermore, no pathological changes were observed in the major organs of the treated mice, indicating minimal systemic toxicity.	[75]
Polyethylene glycol-Gly-Leu-Phe-Gly-Lys(C16)2),	DOX	Cathepsin B	HepG2 Cells	In vitro release studies demonstrated approximately 70% drug release after 70 h, along with cell-specific anticancer activity.	[91]
Arg-His-(Gly-Phe-Lue-Gly)3, RH-(GFLG)3).	DOX	Cathepsin B	HeLa and SW480	DOX-loaded self-assembled nanoparticles exhibited enhanced antitumor efficacy in 3D spheroid cell models and zebrafish models compared to free DOX.	[92]
CPT-LVFFGGGG-PEG-RGD	CPT	Cathepsin B	Both in vitro and in vivo studies were conducted using HeLa cells.	The system exhibited an IC50 value of 4.3 μM and demonstrated approximately 3.9-fold higher tumor-specific accumulation.	[93]
(NPhe)4GGGGk(AZT)y(p)-OH	Azidovudine	Phosphatase enzymes	In vitro NCTC 929 cells	The system exhibited a higher plasma peak concentration (7500 ng mL^−1^), which was approximately 12.5-fold greater than that of the chemically conjugated drug.	[94]
POPE-azobenzene-PEG, iRGD conjugated DSPE	Gemcitabine	Hypoxia	In vitro studies and tumor spheroid assays were performed using BxPC-3 cancer cells	Under hypoxic conditions, the system reduced the viability of pancreatic cancer spheroids to approximately 35%.	[97]
mPEG-Azo-PAsp-IM	Ce6	hypoxia	Both in vitro and in vivo studies were conducted using HeLa and PC-3 cells.	In vivo studies demonstrated substantial tumor volume reduction after 16 days of treatment, along with decreased HIF-1α expression.	[98]
PEG-Azo-PLGA and DATAT-PEGPLGA (8:2)	Ce6 and TPZ	Hypoxia/pH	4T1 in vitro and in vivo	he system generated ROS and, upon laser irradiation, achieved an 86.6% tumor inhibition rate. Furthermore, hematoxylin and eosin (H&E) staining of major organ sections revealed negligible tissue damage across all treatment groups, indicating minimal systemic toxicity.	[99]

## Data Availability

The authors confirm that no new data were generated or analyzed in this study.

## References

[1] FitzGerald G. (2007). The devil’s doctor: Paracelsus and the world of Renaissance magic and science. J. Clin. Investig..

[2] Wang Z., Sun X., Sun M., Wang C., Yang L. (2025). Game Changers: Blockbuster Small-Molecule Drugs Approved by the FDA in 2024. Pharmaceuticals.

[3] Liu B., Zhou H., Tan L., Siu K.T.H., Guan X.-Y. (2024). Exploring Treatment Options in Cancer: Tumor Treatment Strategies. Signal Transduct. Target. Ther..

[4] Park H., Otte A., Park K. (2021). Evolution of Drug Delivery Systems: From 1950 to 2020 and beyond. J. Control. Release.

[5] Luo X., Chen H., Song Y., Qin Z., Xu L., He N., Tan Y., Dessie W. (2023). Advancements, challenges and future perspectives on peptide-based drugs: Focus on antimicrobial peptides. Eur. J. Pharm. Sci..

[6] Yu D., Han N., Son H., Kim S.J., Kweon S. (2026). Functional peptide-based biomaterials for pharmaceutical application: Sequences, mechanisms, and optimization strategies. J. Funct. Biomater..

[7] Rosson E., Lux F., David L., Godfrin Y., Tillement O., Thomas E. (2025). Focus on therapeutic peptides and their delivery. Int. J. Pharm..

[8] Berillo D., Yeskendir A., Zharkinbekov Z., Raziyeva K., Saparov A. (2021). Peptide-based drug delivery systems. Medicina.

[9] Ghosh D., Peng X., Leal J., Mohanty R. (2018). Peptides as drug delivery vehicles across biological barriers. J. Pharm. Investig..

[10] Yatvin M.B., Kreutz W., Horwitz B.A., Shinitzky M. (1980). pH-sensitive liposomes: Possible clinical implications. Science.

[11] Zhang F., Li J., Gao Y., Wei Y., Wen D., Wang Z. (2025). Stimulus switches for spatial-temporal precision in therapeutic delivery systems. Nano Res..

[12] Edelman E.R., Kost J., Bobeck H., Langer R. (1985). Regulation of drug release from polymer matrices by oscillating magnetic fields. J. Biomed. Mater. Res..

[13] Okano T., Bae Y.H., Jacobs H., Kim S.W. (1990). Thermally on–off switching polymers for drug permeation and release. J. Control. Release.

[14] Miyazaki S., Yokouchi C., Takada M. (1988). External control of drug release: Controlled release of insulin from a hydrophilic polymer implant by ultrasound irradiation in diabetic rats. J. Pharm. Pharmacol..

[15] Dreher M.R., Raucher D., Balu N., Colvin O.M., Ludeman S.M., Chilkoti A. (2003). Evaluation of an elastin-like polypeptide–doxorubicin conjugate for cancer therapy. J. Control. Release.

[16] Al Musaimi O., Ng K.W., Gavva V., Mercado-Valenzo O.M., Haroon H.B., William D.R. (2024). Elastin-Derived Peptide-Based Hydrogels as a Potential Drug Delivery System. Gels.

[17] Al-Ahmady Z.S., Al-Jamal W.T., Bossche J.V., Bui T.T., Drake A.F., Mason A.J., Kostarelos K. (2012). Lipid-peptide vesicle nanoscale hybrids for triggered drug release by mild hyperthermia in vitro and in vivo. ACS Nano.

[18] Cheng Y., He C., Ding J., Xiao C., Zhuang X., Chen X. (2013). Thermosensitive hydrogels based on polypeptides for localized and sustained delivery of anticancer drugs. Biomaterials.

[19] Kim M.S., Lee D.-W., Park K., Park S.-J., Choi E.-J., Park E.S., Kim H.R. (2014). Temperature-triggered tumor-specific delivery of anticancer agents by cRGD-conjugated thermosensitive liposomes. Colloids Surf. B Biointerfaces.

[20] Ryu J.S., Raucher D. (2014). Anti-tumor efficacy of a therapeutic peptide based on thermo-responsive elastin-like polypeptide in combination with gemcitabine. Cancer Lett..

[21] Zhang Z., Zhou S., Wang X., Liang R., Sheng X., Zhu Y., Huang L., Zhou B., Zhong M. (2023). Synthesis of pH/temperature-sensitive gelatin/peptide composite hydrogels and its characterization for potential controlled drug release applications. Mater. Today Commun..

[22] Yang Y., Yang Y.F., Xie X.Y., Cai X.S., Wang Z.Y., Gong W., Zhang H., Li Y., Mei X.G. (2015). A near-infrared two-photon-sensitive peptide-mediated liposomal delivery system. Colloids Surf. B Biointerfaces.

[23] Gandioso A., Cano M., Massaguer A., Marchán V. (2016). A green light-triggerable RGD peptide for photocontrolled targeted drug delivery: Synthesis and photolysis studies. J. Org. Chem..

[24] Kumar S., Allard J.-F., Morris D., Dory Y.L., Lepage M., Zhao Y. (2012). Near-infrared light sensitive polypeptide block copolymer micelles for drug delivery. J. Mater. Chem..

[25] Roth-Konforti M.E., Comune M., Halperin-Sternfeld M., Grigoriants I., Shabat D., Adler-Abramovich L. (2018). UV light-responsive peptide-based supramolecular hydrogel for controlled drug delivery. Macromol. Rapid Commun..

[26] Yin L., Tang H., Kim K.H., Zheng N., Song Z., Gabrielson N.P., Lu H., Cheng J. (2013). Light-responsive helical polypeptides capable of reducing toxicity and unpacking DNA: Toward nonviral gene delivery. Angew. Chem. Int. Ed..

[27] Mu R., Gu G., Wang X., Wang R., Wei G. (2026). Biometallic peptide-drug conjugates in photo-crosslinkable hydrogels enable combined photothermal-chemotherapy against breast cancer. J. Nanobiotechnol..

[28] Wei P., Cornel E.J., Du J. (2021). Ultrasound-responsive polymer-based drug delivery systems. Drug Deliv. Transl. Res..

[29] Barmin R.A., Moosavifar M., Dasgupta A., Herrmann A., Kiessling F., Pallares R.M., Lammers T. (2023). Polymeric materials for ultrasound imaging and therapy. Chem. Sci..

[30] Medina S.H., Michie M.S., Miller S.E., Schnermann M.J., Schneider J.P. (2017). Fluorous phase-directed peptide assembly affords nano-peptisomes capable of ultrasound-triggered cellular delivery. Angew. Chem. Int. Ed..

[31] Han H., Lee H., Kim K., Kim H. (2017). Effect of high intensity focused ultrasound (HIFU) in conjunction with a nanomedicines-microbubble complex for enhanced drug delivery. J. Control. Release.

[32] Fan J., Li R., Wang H., He X., Nguyen T.P., Letteri R.A., Zou J., Wooley K.L. (2017). Multi-responsive polypeptide hydrogels derived from N-carboxyanhydride terpolymerizations for delivery of nonsteroidal anti-inflammatory drugs. Org. Biomol. Chem..

[33] Sun M., Yue T., Wang C., Fan Z., Gazit E., Du J. (2022). Ultrasound-responsive peptide nanogels to balance conflicting requirements for deep tumor penetration and prolonged blood circulation. ACS Nano.

[34] Jiang W., Cheng C., Qiu X., Chen L., Guo X., Luo Y., Wang J., Wang J., Xie Z., Li P. (2023). Peptide supramolecular assembly-instructed in situ self-aggregation for stratified targeting sonodynamic therapy enhancement of AIE luminogens. Adv. Sci..

[35] Song B.-L., Wang J.-Q., Zhang G.-X., Yi N.-B., Zhang Y.-J., Zhou L., Guan Y.-H., Zhang X.-H., Zheng W.-F., Qiao Z.-Y. (2024). A coupling-induced assembly strategy for constructing artificial shell on mitochondria in living cells. Angew. Chem. Int. Ed..

[36] Chen Y., Sun H., Li Y., Han X., Yang Y., Chen Z., Zhao X., Qian Y., Liu X., Zhou F. (2025). Magnetic nanomaterials for hyperthermia-based therapy and controlled drug delivery. Bioact. Mater..

[37] Manescu Paltanea V., Antoniac I., Paltanea G., Nemoianu I.V., Mohan A.G., Antoniac A., Rau J.V., Laptoiu S.A., Mihai P., Gavrila H. (2024). Magnetic hyperthermia in glioblastoma multiforme treatment. Int. J. Mol. Sci..

[38] Rodrigues R.O., Baldi G., Doumett S., Garcia-Hevia L., Gallo J., Bañobre-López M., Dražić G., Calhelha R.C., Ferreira I.C.F.R., Lima R. (2018). Multifunctional graphene-based magnetic nanocarriers for combined hyperthermia and dual stimuli-responsive drug delivery. Mater. Sci. Eng. C Mater. Biol. Appl..

[39] Kuskov A.N., Thrapsanioti L.-N., Kukovyakina E., Yagolovich A., Vlaskina E., Tzanakakis P., Berdiaki A., Nikitovic D. (2026). Peptide-functionalized iron oxide nanoparticles for cancer therapy: Targeting strategies, mechanisms, and translational opportunities. Molecules.

[40] Scarberry K.E., Dickerson E.B., McDonald J.F., Zhang Z.J. (2008). Magnetic nanoparticle-peptide conjugates for in vitro and in vivo targeting and extraction of cancer cells. J. Am. Chem. Soc..

[41] Hauser A.K., Anderson K.W., Hilt J.Z. (2016). Peptide conjugated magnetic nanoparticles for magnetically mediated energy delivery to lung cancer cells. Nanomedicine.

[42] Ruan L., Chen W., Wang R., Lu J., Zink J.I. (2019). Magnetically stimulated drug release using nanoparticles capped by self-assembling peptides. ACS Appl. Mater. Interfaces.

[43] Lim Z.W., Varma V.B., Ramanujan R.V., Miserez A. (2020). Magnetically responsive peptide coacervates for dual hyperthermia and chemotherapy treatments of liver cancer. Acta Biomater..

[44] Mañas-Torres M.C., Gila-Vilchez C., Vazquez-Perez F.J., Kuzhir P., Momier D., Scimeca J.-C., Borderie A., Goracci M., Burel-Vandenbos F., Blanco-Elices C. (2021). Injectable magnetic-responsive short-peptide supramolecular hydrogels: Ex vivo and in vivo evaluation. ACS Appl. Mater. Interfaces.

[45] Wang W., Li F., Li S., Hu Y., Xu M., Zhang Y., Khan M.I., Wang S., Wu M., Ding W. (2021). M2 macrophage-targeted iron oxide nanoparticles for magnetic resonance image-guided magnetic hyperthermia therapy. J. Mater. Sci. Technol..

[46] Zhou S., Tsutsumiuchi K., Imai R., Miki Y., Kondo A., Nakagawa H., Watanabe K., Ohtsuki T. (2024). In vitro study of tumor-homing peptide-modified magnetic nanoparticles for magnetic hyperthermia. Molecules.

[47] Biswas S., Bhuniya S. (2024). Fluorescence guided activatable cancer theranostics: Its development and prospect. Smart Drug Delivery Systems—Futuristic Window in Cancer Therapy.

[48] Lin H., Wang L., Jiang X., Wang J. (2024). Glutathione dynamics in subcellular compartments and implications for drug development. Curr. Opin. Chem. Biol..

[49] Meng F., Hennink W.E., Zhong Z. (2009). Reduction-sensitive polymers and bioconjugates for biomedical applications. Biomaterials.

[50] Zhao Y., Yuan K., Hu Q., Li D., Liu M., Zhang J., Zheng H., Liu L. (2024). Preparation of GSH-responsive nanoparticles for combined chemo-photothermal therapy codelivering 6-MP and Ce6. J. Drug Deliv. Sci. Technol..

[51] Liu F.-J., Wang J., Qin Y., Huang B., Liu C., Zhang H., Yu C.-Y., We H. (2025). One-pot synthesis of enzyme and GSH dual-responsive zwitterionic copolymers with cross-linked shells for enhanced anticancer drug delivery. ACS Appl. Polym. Mater..

[52] Ishola B.O., Rahaman K.A., Razzak S.A., Rumon M.M.H., Shakil M.S., Uddin S. (2026). Advanced mechanisms of polymer-based drug delivery systems for clinical applications. RSC Pharm..

[53] Chen J., Ding J., Wang Y., Cheng J., Ji S., Zhuang X., Chen X. (2017). Sequentially responsive shell-stacked nanoparticles for deep penetration into solid tumors. Adv. Mater..

[54] Guo H., Li F., Xu W., Chen J., Hou Y., Wang C., Ding J., Chen X. (2018). Mucoadhesive cationic polypeptide nanogel with enhanced penetration for efficient intravesical chemotherapy of bladder cancer. Adv. Sci..

[55] Halder S., Das T., Kushwaha R., Misra A.K., Jana K., Das D. (2025). Targeted and precise drug delivery using a glutathione-responsive ultra-short peptide-based injectable hydrogel as a breast cancer cure. Mater. Horiz..

[56] Podder A., Joseph M.M., Biswas S., Samanta S., Maiti K.K., Bhuniya S. (2021). Amphiphilic fluorescent probe self-encored in plasma to detect pH fluctuations in cancer cell membranes. Chem. Commun..

[57] Singh J., Nayak P. (2023). pH-responsive polymers for drug delivery: Trends and opportunities. J. Polym. Sci..

[58] Zhuo S., Zhang F., Yu J., Zhang X., Yang G., Liu X. (2020). pH-sensitive biomaterials for drug delivery. Molecules.

[59] Bami M.S., Estabragh M.A.R., Khazaeli P., Ohadi M., Dehghannoudeh G. (2022). pH-responsive drug delivery systems as intelligent carriers for targeted drug therapy: Brief history, properties, synthesis, mechanism and application. J. Drug Deliv. Sci. Technol..

[60] Moyer T.J., Finbloom J.A., Chen F., Toft D.J., Cryns V.L., Stupp S.I. (2014). pH and amphiphilic structure direct supramolecular behavior in biofunctional assemblies. J. Am. Chem. Soc..

[61] Zhu Y., Wang L., Li Y., Huang Z., Luo S., He Y., Han H.F., Wu J., Ge L. (2020). Injectable pH and redox dual-responsive hydrogels based on self-assembled peptides for anti-tumor drug delivery. Biomater. Sci..

[62] Liu Y., Ran Y., Ge Y., Raza F., Li S., Zafar H., Wu Y., Paiva-Santos A.C., Yu C., Sun M. (2022). pH-sensitive peptide hydrogels as a combination drug delivery system for cancer treatment. Pharmaceutics.

[63] Xue W., Trital A., Shen J., Wang L., Chen S. (2020). A zwitterionic polypeptide-based nanodrug augments pH-triggered tumor targeting via prolonging circulation time and accelerating cellular internalization. ACS Appl. Mater. Interfaces.

[64] Fan W., Chen M., Raza F., Zafar H., Jahan F., Chen Y., Ge L., Yang M., Wu Y. (2024). pH-Sensitive Peptide Hydrogel Encapsulating the Anti-Angiogenesis Drug Conbercept and Chemotherapeutic Drug DOX as a Combination Therapy for Retinoblastoma. Mater. Adv..

[65] Chang L., Ran K., Wu F., Tian Y., Wang Y., Liu L., Wu X., Ouyang X., Li B., Ba Z. (2025). A new short pH-responsive anticancer peptide derived by intramolecular charge shielding strategy. Eur. J. Med. Chem..

[66] Auten R.L., Davis J.M. (2009). Oxygen toxicity and reactive oxygen species: The devil is in the details. Pediatr. Res..

[67] Weinstain R., Savariar E.N., Felsen C.N., Tsien R.Y. (2014). In vivo targeting of hydrogen peroxide by activatable cell-penetrating peptides. J. Am. Chem. Soc..

[68] Kita M., Yamamoto J., Morisaki T., Komiya C., Inokuma T., Miyamoto L., Tsuchiya K., Shigenaga A., Otaka A. (2015). Design and synthesis of a hydrogen peroxide-responsive amino acid that induces peptide bond cleavage after exposure to hydrogen peroxide. Tetrahedron Lett..

[69] Liang J., Liu B. (2016). ROS-responsive drug delivery systems. Bioeng. Transl. Med..

[70] Keng C.L., Lin Y.C., Tseng W.L., Lu C.Y. (2017). Design of peptide-based probes for the microscale detection of reactive oxygen species. Anal. Chem..

[71] Hao Y., Song K., Tan X., Ren L., Guo X., Zhou C., Li H., Wen J., Meng Y., Lin M. (2022). Reactive oxygen species-responsive polypeptide drug delivery system targeted activated hepatic stellate cells to ameliorate liver fibrosis. ACS Nano.

[72] Hara Y., Yoshizawa K., Yaguchi A., Hiramatsu H., Uchida N., Muraoka T. (2024). ROS-responsive methionine-containing amphiphilic peptides impart enzyme-triggered phase transition and antioxidant cell protection. Biomacromolecules.

[73] De Sciscio M.L., Centola F., Saporiti S., D’Abramo M. (2025). Dissecting Methionine Oxidation by Hydrogen Peroxide in Proteins: Thermodynamics, Kinetics, and Susceptibility Descriptors. J. Chem. Inf. Model..

[74] Edirisinghe D.I.U., Singh A., Seitz M.P., Esmaeili A.J., Orado T.K., Herrero E., Jankowski J., Kairuki P.K., Marshall L.R., Jain E. (2025). A Peptide Hydrogel Responsive to Reactive Oxygen Species and pH for the Protection and Sustained Delivery of Insulin-like Growth Factor 1 in Osteoarthritis Treatment. ACS Appl. Bio Mater..

[75] Zhang S., Wei Q., Lv J., Gong X., Wang B., Ren Z., Wei C., Guo Z., Li J.L. (2026). ROS-Activated Peptide-Based Prodrug for Chemoselective Covalent Targeting in Cancer Cells. J. Med. Chem..

[76] Mocellin S., Bronte V., Nitti D. (2007). Nitric oxide, a double-edged sword in cancer biology: Searching for therapeutic opportunities. Med. Res. Rev..

[77] Zhao H., Zhang Y., Fu X., Chen C., Khattak S., Wang H. (2023). The double-edged sword role of hydrogen sulfide in hepatocellular carcinoma. Front. Pharmacol..

[78] Liu R., Xu M., Yan Q. (2020). Nitric Oxide-Biosignal-Responsive Polypeptide Nanofilaments. ACS Macro Lett..

[79] Duan S., Cai S., Yang Q., Forrest M.L. (2012). Multi-arm polymeric nanocarrier as a nitric oxide delivery platform for chemotherapy of head and neck squamous cell carcinoma. Biomaterials.

[80] Liu R., Xu B., Ma Z., Ye H., Guan X., Ke Y., Xiang Z., Shi Q. (2022). Controlled release of nitric oxide for enhanced tumor drug delivery and reduction of thrombosis risk. RSC Adv..

[81] Li Z., Tousian B., Zaiden M., Sarkar I., Vu C., Wang Y., Bitton R., Matson J.B. (2025). Cellular Uptake and Antioxidant Activity of H2S-Releasing Tetrapeptide Supramolecular Polymer Nanostructures. ACS Mater. Lett..

[82] Longchamp A., Kaur K., Macabrey D., Dubuis C., Corpataux J.M., Déglise S., Matson J.B., Allagnat F. (2019). Hydrogen Sulfide-Releasing Peptide Hydrogel Limits the Development of Intimal Hyperplasia in Human Vein Segments. Acta Biomater..

[83] Carter J.M., Qian Y., Foster J.C., Matson J.B. (2015). Peptide-Based Hydrogen Sulphide-Releasing Gels. Chem. Commun..

[84] Ali R., Hameed R., Chauhan D., Sen S., Wahajuddin M., Nazir A., Verma S. (2022). Multiple Actions of H2S-Releasing Peptides in Human β-Amyloid Expressing *C. elegans*. ACS Chem. Neurosci..

[85] Podder A., Senapati S., Maiti P., Kamalraj D., Jaffer S.S., Khatun S., Bhuniya S. (2018). A ‘Turn-On’ Fluorescent Probe for Lysosomal Phosphatase: A Comparative Study for Labeling of Cancer Cells. J. Mater. Chem. B.

[86] Bobba K.N., Won M., Shim I., Valusamy N., Yang Z., Qu J., Kim J.S., Bhuniya S. (2017). A BODIPY-Based Two-Photon Fluorescent Probe Validates Tyrosinase Activity in Live Cells. Chem. Commun..

[87] Liu H.W., Chen L., Xu C., Li Z., Zhang H., Zhang X.B., Tan W. (2018). Recent progresses in small-molecule enzymatic fluorescent probes for cancer imaging. Chem. Soc. Rev..

[88] Kaniewska K., Mackiewicz M., Smutok O., Gonchar M., Katz E., Karbarz M. (2024). Enzymatically Triggered Drug Release from Microgels Controlled by Glucose Concentration. ACS Biomater. Sci. Eng..

[89] Wei Y., Lv J., Zhu S., Wang S., Su J., Xu C. (2024). Enzyme-Responsive Liposomes for Controlled Drug Release. Drug Discov. Today.

[90] Fouladi F., Steffen K.J., Mallik S. (2017). Enzyme-Responsive Liposomes for the Delivery of Anticancer Drugs. Bioconjug. Chem..

[91] Lee S., Song S.J., Lee J., Ha T.H., Choi J.S. (2020). Cathepsin B-Responsive Liposomes for Controlled Anticancer Drug Delivery in Hep G2 Cells. Pharmaceutics.

[92] Song S.J., Choi J.S. (2022). Enzyme-Responsive Amphiphilic Peptide Nanoparticles for Biocompatible and Efficient Drug Delivery. Pharmaceutics.

[93] Cheng D.B., Wang D., Gao Y.J., Wang L., Qiao Z.Y., Wang H. (2019). Autocatalytic Morphology Transformation Platform for Targeted Drug Accumulation. J. Am. Chem. Soc..

[94] Coulter S.M., Pentlavalli S., An Y., Vora L.K., Cross E.R., Moore J.V., Sun H., Schweins R., McCarthy H.O., Laverty G. (2024). In Situ Forming, Enzyme-Responsive Peptoid–Peptide Hydrogels: An Advanced Long-Acting Injectable Drug Delivery System. J. Am. Chem. Soc..

[95] Bhuniya S., Vrettos I.E. (2024). Hypoxia-Activated Theragnostic Prodrugs (HATPs): Current State and Future Perspectives. Pharmaceutics.

[96] Zhao Y.Q., Biswas S., Chen Q., Jia M., Zhou Y., Bhuniya S. (2021). Direct Readout Hypoxia Tumor Suppression In Vivo through NIR-Theranostic Activation. ACS Appl. Bio Mater..

[97] Kulkarni P., Haldar M.K., Katti P., Dawes C., You S., Choi Y., Mallik S. (2016). Hypoxia-Responsive, Tumor Penetrating Lipid Nanoparticles for Delivery of Chemotherapeutics to Pancreatic Cancer Cell Spheroids. Bioconjug. Chem..

[98] Li J., Meng X., Jian D., Lu D., Zhang X., Chen Y., Zhu J., Fan A., Ding D., Kong D. (2018). Multifunctional Micelles Dually Responsive to Hypoxia and Singlet Oxygen: Enhanced Photodynamic Therapy via Interactively Triggered Photosensitizer Delivery. ACS Appl. Mater. Interfaces.

[99] Ihsanullah K.M., Kumar B.N., Zhao Y., Muhammad H., Liu Y., Wang L., Liu H., Jiang W. (2020). Stepwise-Activatable Hypoxia-Triggered Nanocarrier-Based Photodynamic Therapy for Effective Synergistic Bioreductive Chemotherapy. Biomaterials.

[100] Zheng W., Yao S.-Y., Hu H., Chen X., Qian Z., Liu W., Zhu Y., Mao Z., Guo D.-S., Gao C. (2024). Hypoxia-responsive calixarene-grafted self-assembled peptide hydrogel for inflammation suppression in ischemic stroke. Nano Today.

[101] Xiao W., Zeng X., Lin H., Han K., Jia H.-Z., Zhang X.-Z. (2015). Dual Stimuli-Responsive Multi-Drug Delivery System for the Individually Controlled Release of Anti-Cancer Drugs. Chem. Commun..

[102] Zhang X., Yin T., Wang S., Hao Z., He Y., Li C., Zhao Q., He H., Gao D. (2019). Dual stimuli-responsive peptide-based palladium nano-lychee spheres for synergistic antitumor therapy. ACS Biomater. Sci. Eng..

[103] Li Q., Fu D., Zhang J., Yan H., Wang H., Niu B., Guo R., Liu Y. (2021). Dual stimuli-responsive polypeptide-calcium phosphate hybrid nanoparticles for co-delivery of multiple drugs in cancer therapy. Colloids Surf. B Biointerfaces.

[104] Chen J., Song Y., Yang W., Guo J., Zhang S., Wan D., Liu Y., Pan J. (2023). Enzyme and Reduction Dual-Responsive Peptide Micelles as Nanocarriers for Smart Drug Delivery. ACS Appl. Nano Mater..

[105] Jiao X., Han M., Wang Z., Min J., Su R., Tie H., Qi W., Wang Y. (2026). Modular Design of Dual-Targeted, Enzyme-Responsive Peptide Carriers for Gene Drug Delivery. Nanoscale Horiz..

[106] Huang Y., Hsu J.C., Koo H., Cormode D.P. (2022). Repurposing Ferumoxytol: Diagnostic and Therapeutic Applications of an FDA-Approved Nanoparticle. Theranostics.

[107] Lai L., Han X., Tang Y., Zhou J., Cui W. (2026). Advances in Ultrasound-Assisted Drug Delivery and Clinical Application. Ultrason. Sonochem..

[108] Bobba K.N., Binoy A., Koo S., Podder A., Mishra A., Mishra N., Kim J.S., Bhuniya S. (2019). Direct Readout Protonophore-Induced Selective Uncoupling and Dysfunction of Individual Mitochondria within Cancer Cells. Chem. Commun..

[109] Bobba K.N., Saranya G., Sujai P.T., Joseph M.M., Velusamy N., Podder A., Maiti K.K., Bhuniya S. (2019). Endogenous H_2_S-Assisted Cancer Cell-Specific Activation of Theranostic with Emission Readout. ACS Appl. Bio Mater..

[110] Wu K., Sun L., Wu H., Zheng X., Xue Y., Zhang Z., Ma S., Zhao C., Liu Y., Gu X. (2025). H_2_S-Activated Theranostic Platform for Precise Photoablation of Colorectal Tumors with NIR-II Dual-Modal Phototherapy. J. Med. Chem..

[111] Niu H., Wang S., Liu Y., Ma N., Cheng S., Feng B., Jeong H., Yang Y., Wang G., James T.D. (2025). Naphthalimide-Based Type-I Nano-Photosensitizers for Enhanced Antitumor Photodynamic Therapy: H_2_S Synergistically Regulates PeT and Self-Assembly. Angew. Chem. Int. Ed..

